# Exploring effective biomarkers and potential immune related gene in small cell lung cancer

**DOI:** 10.1038/s41598-024-58454-4

**Published:** 2024-03-31

**Authors:** Yang Yunchu, Akihiko Miyanaga, Kuniko Matsuda, Koichiro Kamio, Masahiro Seike

**Affiliations:** Department of Pulmonary Medicine and Oncology, Graduate School of Medicine, Nippon Medical School, Tokyo, Japan

**Keywords:** Lung cancer, Oncogenes, Tumour biomarkers

## Abstract

Small cell lung cancer (SCLC) is well known as a highly malignant neuroendocrine tumor. Immunotherapy combined with chemotherapy has become a standard treatment for extensive SCLC. However, since most patients quickly develop resistance and relapse, finding new therapeutic targets for SCLC is important. We obtained four microarray datasets from the Gene Expression Omnibus database and screened differentially expressed genes by two methods: batch correction and “RobustRankAggregation”. After the establishment of a protein–protein interaction network through Cytoscape, seven hub genes (AURKB, BIRC5, TOP2A, TYMS, PCNA, UBE2C, and AURKA) with high expression in SCLC samples were obtained by eight CytoHubba algorithms. The Least Absolute Shrinkage and Selection Operator regression and the Wilcoxon test were used to analyze the differences in the immune cells’ infiltration between normal and SCLC samples. The contents of seven kinds of immune cells were considered to differ significantly between SCLC samples and normal samples. A negative association was found between BIRC5 and monocytes in the correlation analysis between immune cells and the seven hub genes. The subsequent in vitro validation of experimental results showed that downregulating the expression of BIRC5 by siRNA can promote apoptotic activity of SCLC cells and inhibit their vitality, migration, and invasion. The use of BIRC5 inhibitor inhibited the vitality of SCLC cells and increased their apoptotic activity. BIRC5 may be a novel therapeutic target option for SCLC.

## Introduction

Lung cancer is a malignant tumor developing from the gland or bronchial mucosa of the lung, and poses the biggest threat to people’s health and life. Its incidence rate and mortality are extremely high and growing worldwide^1^. Small cell lung cancer (SCLC) accounts for about 15% of all cases of lung cancer, proliferates quickly, and metastasizes widely in the early stage. Most patients have already experienced systemic metastasis when diagnosed^2^. For many years, platinum plus etoposide combined chemotherapy has been the first-line treatment plan for SCLC^3^. Most SCLC patients initially respond effectively to chemotherapy, but the rapid emergence of chemotherapy resistance significantly affects their prognoses^4^. recently, immune checkpoint inhibitors (ICIs) have been progressively applied in treating SCLC^5,6^. Although their development has improved the selection of SCLC treatment methods, the immunotherapy effect is still limited due to the low expression of Programmed cell death 1 ligand 1 (PD-L1) and the occurrence of immunotherapy resistance. Immune-related adverse events may occur^7^. Therefore, the demand for new biomarkers that can be used for SCLC treatment is obvious.

Bioinformatics analysis is frequently used to look for potential biomarkers for many diseases, including SCLC, due to the quick development of microarray and high-throughput sequencing technologies^8–10^. In this study we sought to identify genes associated with SCLC, especially immunotherapy, by bioinformatics analysis. We obtained differentially expressed genes (DEGs) between SCLC and normal tissues by comprehensive analysis of four microarray datasets from the Gene Expression Omnibus (GEO).

This study not only screened novel and effective SCLC biomarkers, but also provided a new possibility for the treatment of SCLC, especially immunotherapy.

## Materials and methods

### Data collection and preparation

Four gene expression profiles were screened out from the GEO (http://www.ncbi.nlm.nih.gov/geo) database: GSE6044, GSE40275, GSE108055, and GSE149507. The inclusion criteria for the datasets were: (1) the object was human, and featured SCLC samples and normal lung samples; (2) the sample size of the dataset was more than 10. The robust multiarray average (RMA) algorithm in the R package “affy” was used to normalize the raw data and transform the probe expression matrix into a matrix of gene expression. We took the average of the expression data in cases when numerous probes corresponded to the same gene. We used the R package “sva” to eliminate batch effects on merged expression data, to reduce analysis errors.

### Screening of differentially expressed genes (DEGs) in small cell lung cancer

DEGs were obtained through two methods. The Surrogate variable analysis (SVA) method first consolidated all raw data into one matrix and performed batch correction on it, and then analyzed it using the R package “limma”. The other method was to use RobustRankAggregation (RRA) in the R package to integrate the DEGs of each dataset. Finally, we applied Venn diagrams to the results obtained by these two methods and obtained intersecting DEGs. The parameters of the DEGs are: |log fold change (FC)|> 1, and adjusted *p* < 0.05.

### Pathway and functional enrichment analysis

The Kyoto Encyclopedia of Genes and Genomes (KEGG)^11–13^ and Gene Ontology (GO) analyses of DEGs were completed by the R package “clusterProfiler”. The filtering condition was set to: adjusted *p* < 0.05.

### Establishment of protein–protein interaction (PPI) network and hub genes screening

A PPI network was created via STRING (https://string-db.org) database, with a filtering condition of an interaction score greater than 0.7. Eight algorithms taken from the CytoHubba plugin of Cytoscape V3.9.1^14^ were used to score each node gene. We then used the R package “UpSet” to screen the top 60 genes of each algorithm, and obtained hub genes. GSE11969 was used to verify the accuracy of the hub genes. Their diagnostic effect was estimated by the receiver operating characteristic (ROC) curve and the area under curve (AUC). The hub genes logistic regression model used to differentiate between SCLC samples and normal samples was established by the R package “glm”.

### Immune infiltration analysis

The CIBERSORT (Cell-type Identification By Estimating Relative Subsets Of RNA Transcripts) (https://cibersort.stanford.edu) algorithm was utilized to perform immune cells infiltration analysis on a microenvironment of merged data from four datasets. (Information on the proportion of infiltrating immune cells is displayed in Supplementary Info File 1). In addition, the Pearson analysis in the R package was utilized to find the connection between 22 immune cells and hub genes. The analysis and visualization of principal components analysis (PCA) were completed by R packages “FactoMine” and “factoextra”, respectively.

### Cell culture and inhibitors

Human SCLC cell lines SBC3 and SBC5 were purchased from the Japanese Collection of Research Bioresources Cell Bank. SCLC cell line H1048, non-small cell lung cancer (NSCLC) cell lines A549, EBC1, and EREF-LC-KJ, and human lung fibroblasts (HLFs) were purchased from American Type Culture Collection. All cells were cultured with RPMI-1640 (FUJIFILM Wako Pure Chemical Corporation) with 10% fetal bovine serum (Biosera). BIRC5 inhibitor (YM155) was purchased from Selleck Chemicals and used to culture SBC3, SBC5, and H1048 cells at concentrations of 2 nM, 3 nM, and 7 nM for 48 h to inhibit the function of BIRC5.

### Transfection

The small interfering RNA (siRNA) targeting BIRC5 was purchased from Invitrogen with a sequence of Sense (5′-3′): GCAGGUUCCUUAUCUGUCAtt; Antisense (5′-3′): UGACAGAUAAGGAACCUGCag. In accordance with the manufacturer’s guidelines, we used Lipofectamine RNAiMAX (Invitrogen) and Opti-MEM (Gibco) to transiently transfect cells with siRNA. After 24 h, we replaced the culture medium for further cultivation and conducted subsequent experiments.

### RNA isolation and quantitative real-time PCR

Total RNA was isolated from cells using Isogen reagent (Nippon Gene), and its concentration was determined by NanoDrop 2000 (Thermo Fisher Scientific Inc). The THUNDERBIRD Probe qPCR Mix kit (Toyobo) and 7500 Fast Real time PCR instrument (Thermo) were then used for cDNA acquisition and qRT-PCR. The 2 − ΔΔCT method was used to calculate mRNA expression levels. Primers were purchased from Applied Biosystems (GAPDH: 4352934E; BIRC5: Hs00153353).

### Western blot

We extracted total proteins from cells by RIPA lysis buffer (FUJIFILM Wako Pure Chemical Inc) and detected their concentration by protein assay BCA kit (FUJIFILM). The proteins were separated by SDS polyacrylamide gel electrophoresis and moved to a polyvinylidene fluoride membrane. We added 5% skim milk powder (FUJIFILM) in 1 × TBST to block the membrane for 1.5 h, then incubated the membrane overnight with specific primary antibodies at 4 °C. Among them, BIRC5 (Survivin) antibody was purchased from Proteintech Japan; antibodies for PARP, cleaved PARP, caspase3, and β-actin were purchased from Cell Signaling Technology. The next day we washed the membrane three times with 1 × TBST and incubated with HRP conjugated anti-rabbit or anti-mouse secondary antibodies (Southern Biotech) for 1.5 h. Finally, the protein was detected by an Amersham Imager 600 (Thermo).

### Cell proliferation assay

Cells were planted on six-well plates and transfected with non-targeting control or BIRC5 siRNA. The next day, the cells were digested and planted on 96-well plates and continued to be cultured. We added 10% CCK-8 solution (Dojindo) in culture medium at 0 h, 24 h, 48 h, and 72 h after cultivation, then continued to cultivate for 1–2 h. We used Infinite200 PRO (Tecan) to detect the 450 nm optical density (OD) values of each well.

### Transwell cell invasion assay

We planted the non-targeting control and BIRC5 siRNA transfected cells separately on a Transwell upper chamber (Falcon) with a pore size of 8.0 μM, coated with Matrigel® Matrix (Corning) and serum-free medium, then put the upper chamber onto 12-well plates with 10% fetal bovine serum medium for 24 h of further cultivation. We fixed the Transwell membrane with 4% paraformaldehyde for 15 min, then stained with Giemsa’s solution (Merck) and took photographs and counted cells under a light microscope (Olympus IX700).

### Scratch wound healing assay

After 24 h of transfection of cells with siRNA, the cells were digested and planted on six-well plates for a further 24 h of cultivation. We scratched the middle part of each well with a 1 ml sterile pipette tip and removed cell debris. We photographed the scratched areas under the light microscope at 0 and 48 h after the scratch, and compared the changes of the scratched areas.

### Apoptosis detection assay

We planted the cells on six-well plates, added siRNA, and incubated for 48 h. After the cells were digested, we washed them twice with PBS, added 5 μl Annexin V (Nacalai Tesque Inc) and propidium iodide (BioLegend), cultured in the dark for 15 min. A FACSVerse™ flow cytometer (BD Biosciences) was utilized for analysis.

### Statistical analysis

All statistical analyses were completed by R software (version 4.2.3; https://www.r-project.org) and GraphPad Prism (GraphPad Software Inc). The two groups were compared by a Student’s t-test, and comparisons of more than three samples were undertaken using ANOVA (Analysis of Variance) followed by Tukey–Kramer’s test. Values of *p* < 0.05 were regarded as statistically significant (*p < 0.05; **p < 0.01; ***p < 0.001).

## Results

### Identification of DEGs

Two techniques were used to determine DEGs in four microarray datasets, namely RRA and SVA. 1180 genes identified by RRA and the DEGs in each dataset are shown in Supplementary Info File 2.

**Figure 1:**
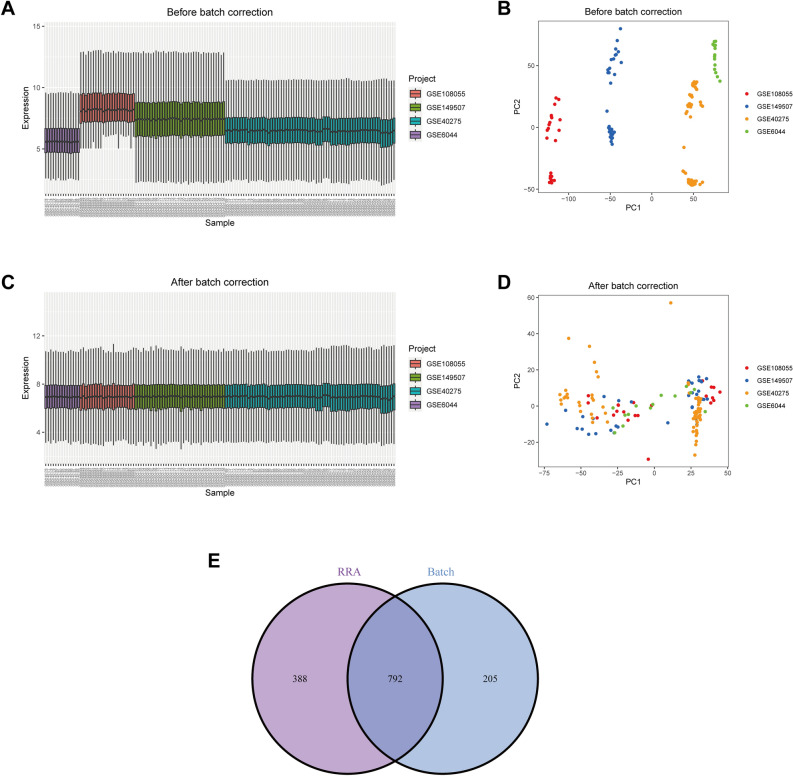
Batch correction of data and determination of DEGs. Box plots and principal component analysis of four datasets before (**A**, **B**) and after (**C**, **D**) correction. (**E**) Intersecting DEGs obtained by two methods.

The data before and after batch correction are shown in Fig. 1A–B and C–D, respectively. The merged data's batch effect has been removed. After difference analysis of the merged data, a total of 997 genes were identified as DEGs (Supplementary Info File 3). After intersecting the results obtained by these two methods, we obtained 792 DEGs (Fig. 1E).

### Function and pathway analysis of DEGs

In order to investigate the biological functions and pathways involved in DEGs, we performed KEGG and GO analyses. The KEGG analysis results indicate that these genes were primarily enriched in “Cell cycle”, “Composition and coagulation cascades”, and “DNA replication” (Fig. S1A–B). The GO analysis results indicated that these genes possessed immune-related functions such as “cell chemotaxis”, “leukocyte chemotaxis”, and “leukocyte migration” (Fig. S1C–D).

### Establishment of PPI network and hub genes screening

**Figure 2:**
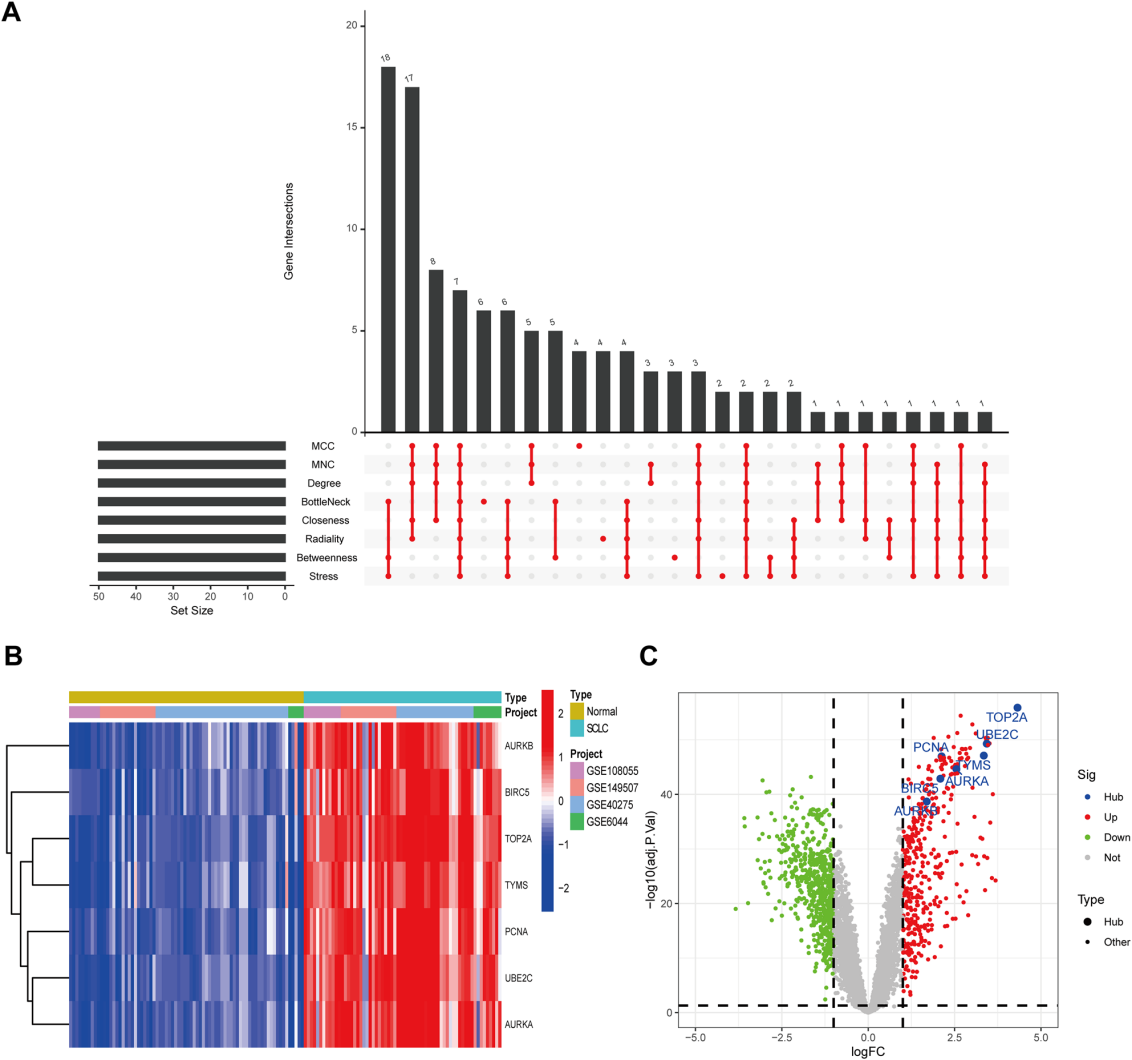
Discovery* o*f hub genes. (**A**) Eight algorithms confirmed seven hub genes. (**B**) Heatmap of hub gene expression in merged data. (**C**) The location of hub genes in volcano maps.

The PPI network of 792 DEGs was established by Cytoscape (Fig. S2), and includes 468 nodes and 1685 interaction pairs (Supplementary Info File 4). Next, we used the R package “UpSet” to obtain seven hub genes (*AURKB*, *BIRC5*, *TOP2A*, *TYMS*, *PCNA*, *UBE2C*, and *AURKA*) through eight algorithms (Fig. 2A–B). The volcano plot obtained after the merger of microarray data indicated that the expression level of all seven genes was greater in SCLC samples than in normal samples (Fig. 2C).

### Verification of hub genes

**Figure 3:**
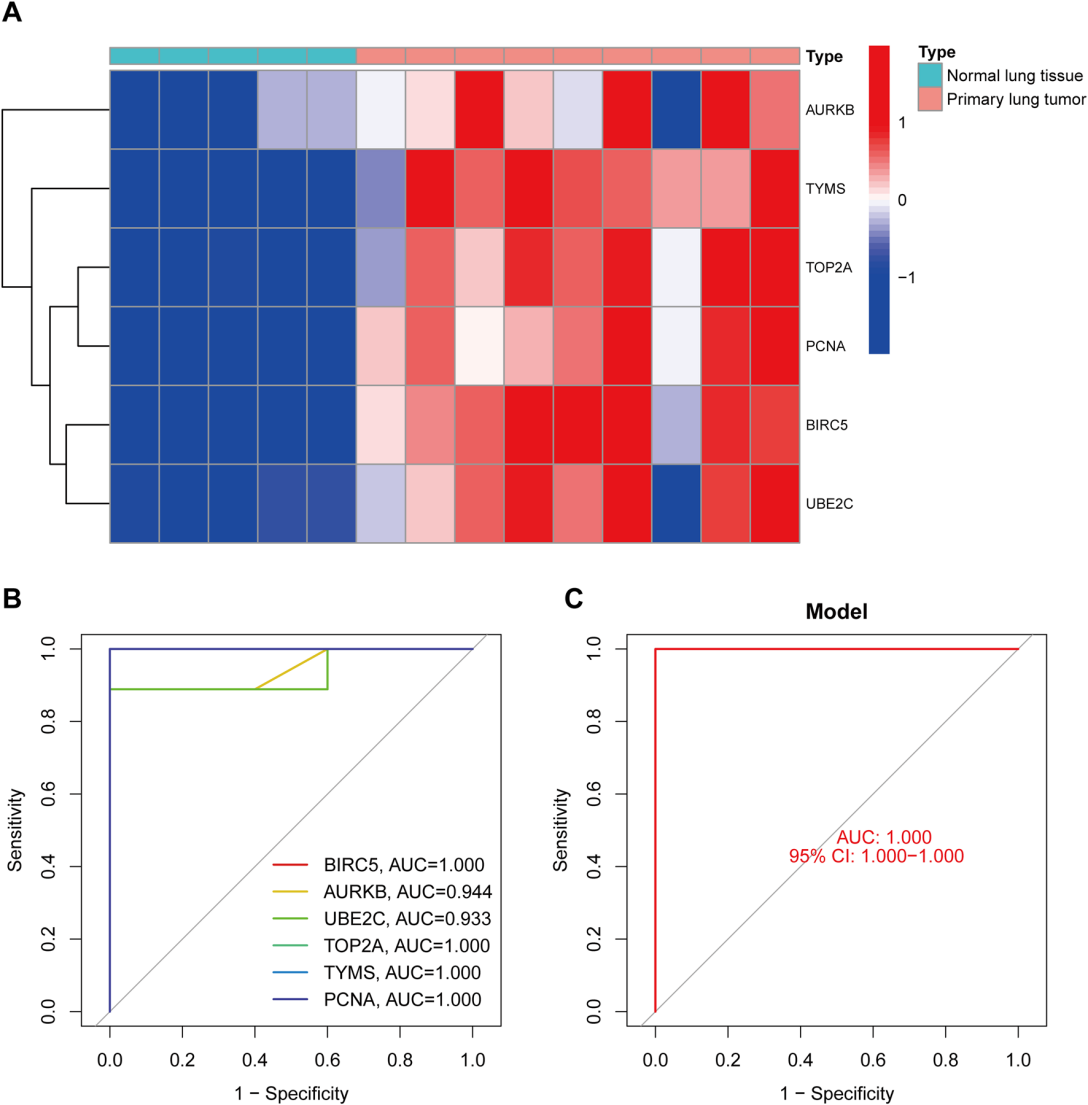
Verification of hub genes in GSE11969*.* (**A**) The expression of *AURKB, BIRC5, TOP2A, TYMS, PCNA,* and *UBE2C* were displayed in the heatmap. (**B**, **C**) ROC curves of hub genes in GSE11969.

To verify the accuracy of this result, we chose another dataset, GSE11969, as our test cohort. The heatmap (Fig. 3A) and violin plot (Fig. S3A–H) show that the expressions level of *AURKB, BIRC5, TOP2A, TYMS, PCNA,* and *UBE2C* were markedly greater in SCLC samples than in normal samples, in accordance with the previous results.

In addition, GSE11969 was used for ROC analysis. The results demonstrated that the area under the ROC curve (AUC) of all six genes was greater than 0.9 (Fig. 3B), while the AUC of the regression model combined with six hub genes was 1 (Fig. 3C). This result also verified the accuracy of these hub genes as biomarkers for SCLC.

### Correlation between hub genes and immune cell infiltration in SCLC

**Figure 4:**
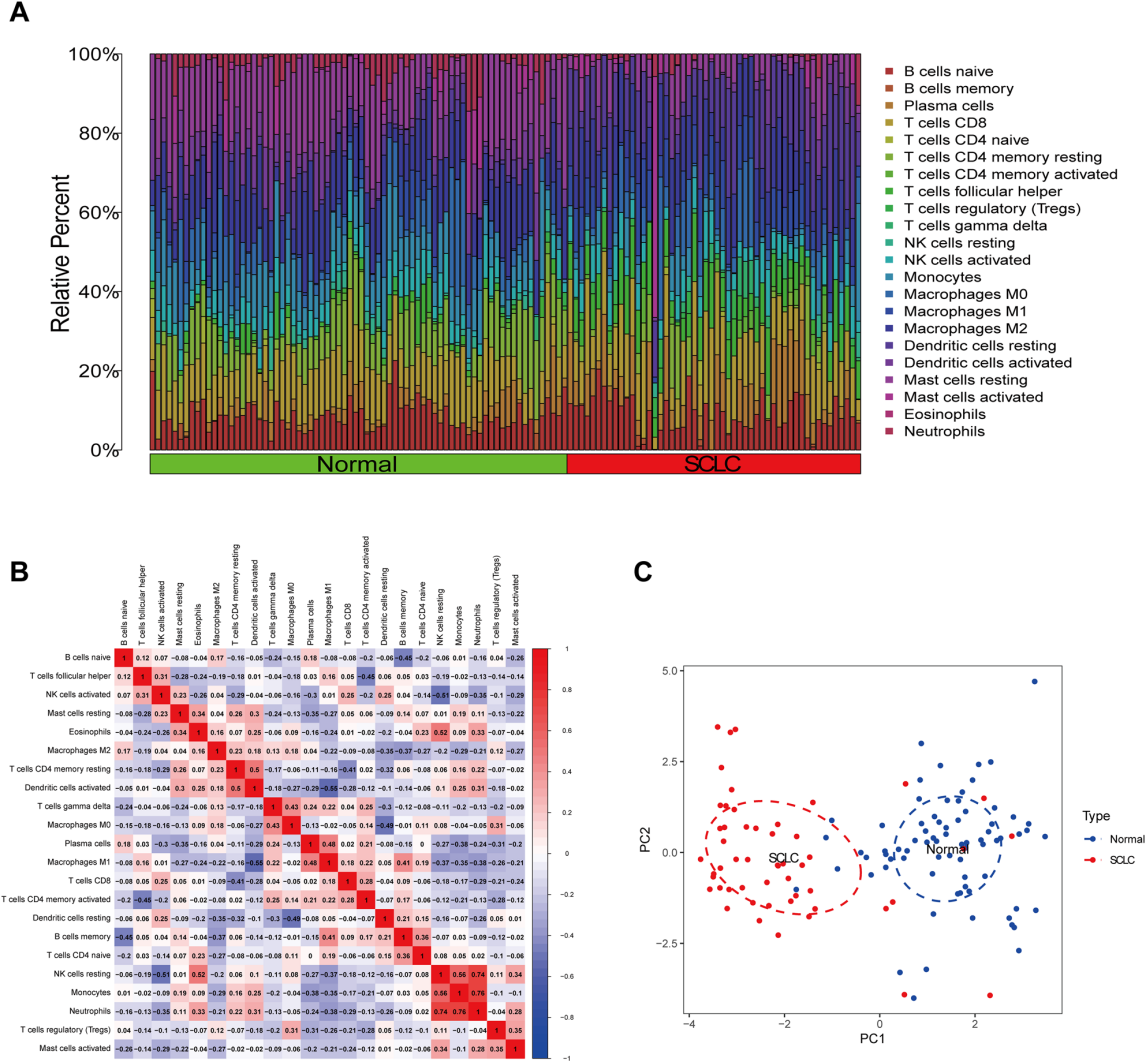
Analyses of SCLC and normal samples’ immune cell infiltration. (**A**) The barplot showed the composition of 22 types of immune cells in each sample. (**B**) The heatmap showed the correlation between 22 types of immune cells in SCLC samples. (**C**) Classification of infiltrating immune cells between SCLC and normal samples through PCA analysis.

Next, we conducted an immune infiltration analysis using batch corrected data. Firstly, the barplot showed the relative percentage of 22 immune cells in each sample (Fig. 4A). We then conducted a correlation analysis of immune cells in SCLC samples. For example, NK cells resting correlated positively with eosinophils, monocytes, and neutrophils. M1 macrophages were positively correlated with plasma cells and B cell memory. Dendritic cells resting correlated negatively with M0 and M2 macrophages (Fig. 4B). Thirdly, based on the infiltration of immune cells, SCLC and normal samples can be completely distinguished through PCA (Fig. 4C).

Two methods, the Wilcoxon test and LASSO regression, were then employed to analyze the various infiltrations of immune cells in SCLC samples and normal samples. The Wilcoxon test identified 13 immune cells (Fig. S4A) and LASSO regression identified seven immune cells (Fig. S4B–C). Finally, we obtained seven intersecting immune cells that significantly affect SCLC immune infiltration (Fig. S4D, Table S1). Among them, the content of T cells CD4 memory activated, T cells follicular helper, M1 macrophages and dendritic cells resting were higher in SCLC samples, while the content of T cells CD4 memory resetting, monocytes, and mast cells resting were higher in normal samples.

### Confirmation of the correlation between BIRC5 and monocytes

**Figure 5:**
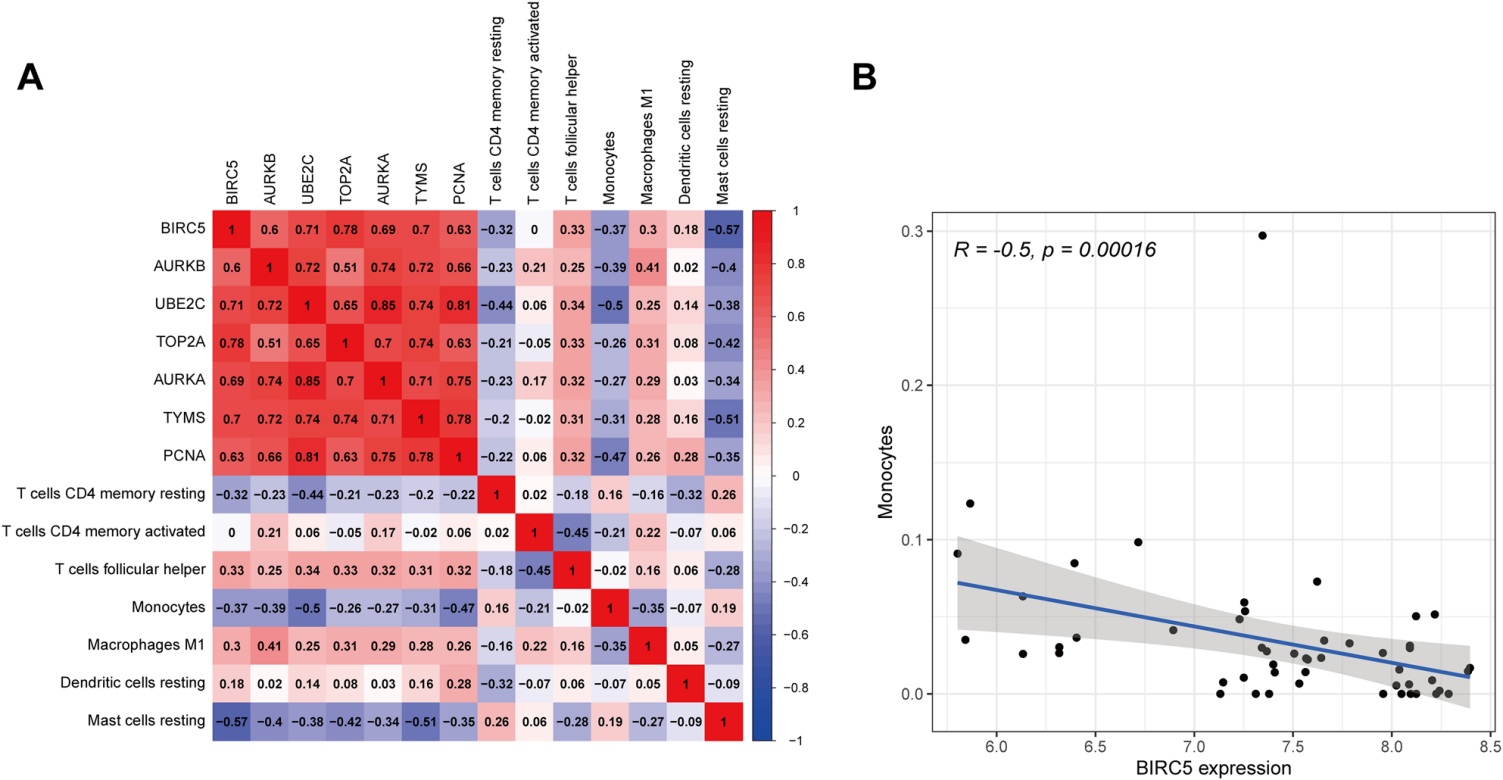
Correlation analysis between hub genes and differential immune cells. (**A**) The relationship between seven hub genes and seven immune cells was displayed in the heatmap. (**B**) BIRC5 was substantially associated with monocytes, |R|> 0.40, *p* < 0.001.

A correlation analysis was then conducted on these seven immune cells and seven hub genes (*AURKB, BIRC5, TOP2A, TYMS, PCNA, UBE2C,* and *AURKA*). The results are shown in Fig. 5A. The hub genes and differential immune cells were analyzed under conditions of |R|> 0.40 and *p* < 0.001. Ultimately, BIRC5 was considered to be negatively correlated with the content of monocytes (R = − 0.5, *p* = 0.00016) (Fig. 5B).

### In vitro validation experiment

**Figure 6:**
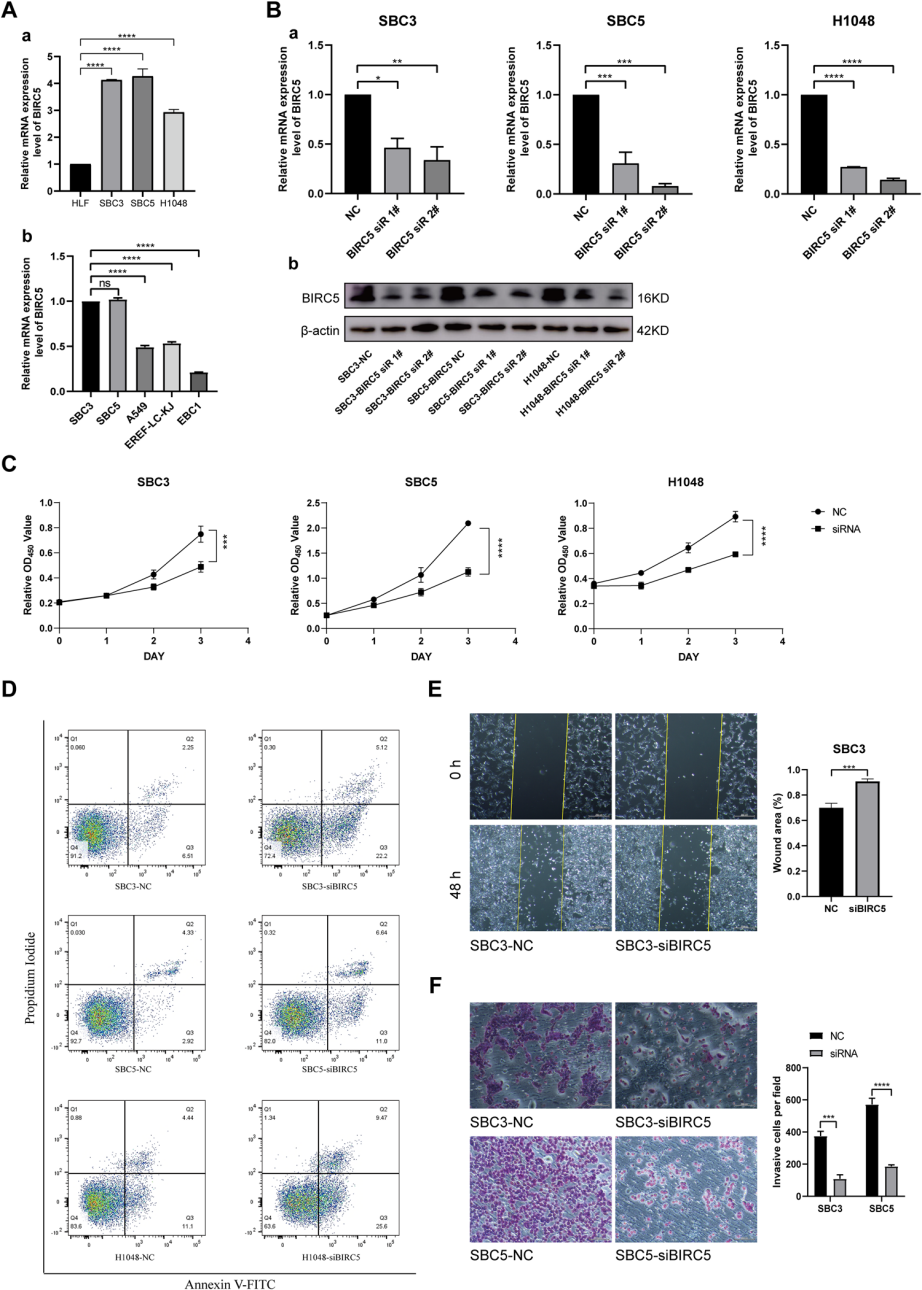
BIRC5 inhibited the apoptosis of SCLC cells and promoted their proliferation, migration, and invasion. (**A**) Validation of BIRC5 expression in SCLC cells, HLF and NSCLC cells by qRT-PCR analysis. (**B**) Verification of RNA knockout effect by qRT-PCR and western blotting (original blots are presented in Supplementary information 2). (**C**) CCK8 cell proliferation assay results. (**D**) Cell apoptosis detected by flow cytometry. (E) *Scratch wound healing assay* results. (**F**) Transwell cell invasion assay results.

We wished to further validate these analysis results. As BIRC5 was the only hub gene believed to be associated with SCLC immune infiltration, we chose BIRC5 for further validation. We found that SCLC cell lines SBC3, SBC5, and H1048 had greater BIRC5 expression than HLFs and non-small cell lung cancer cell lines A549, EREF-LC-KJ, and EBC1 (Fig. 6A). To demonstrate the functional connection between BIRC5 and SCLC, we designed two different BIRC5 siRNAs and transfected three SCLC cell lines: SBC3, SBC5, and H1048 cells. The decreased effect of siBIRC5-2 was higher than that of siBIRC5-1 (Fig. 6B). Therefore, we decided to use siBIRC5-2 for the subsequent experiments. A CCK-8 assay showed that within 72 h after BIRC5 inhibition, the proliferation ability of all three cell lines decreased (Fig. 6C). According to flow cytometry and western blotting, the apoptotic activity of the SCLC cell lines increased after the downregulation of BIRC5 (Figs. 6D, S5A). In addition, scratch wound healing assay indicated that migration activity of SCLC cell lines weakened after the downregulation of BIRC5 (Figs. 6E, S5B). Transwell cell invasion assay that after BIRC5 downregulation, the invasive capacity of SCLC cell lines was greatly reduced (Figs. 6F, S5C). These results suggest that BIRC5 may affect the disease progression of SCLC by promoting cell proliferation, migration, invasion, and reducing cell apoptosis.

**Figure 7:**
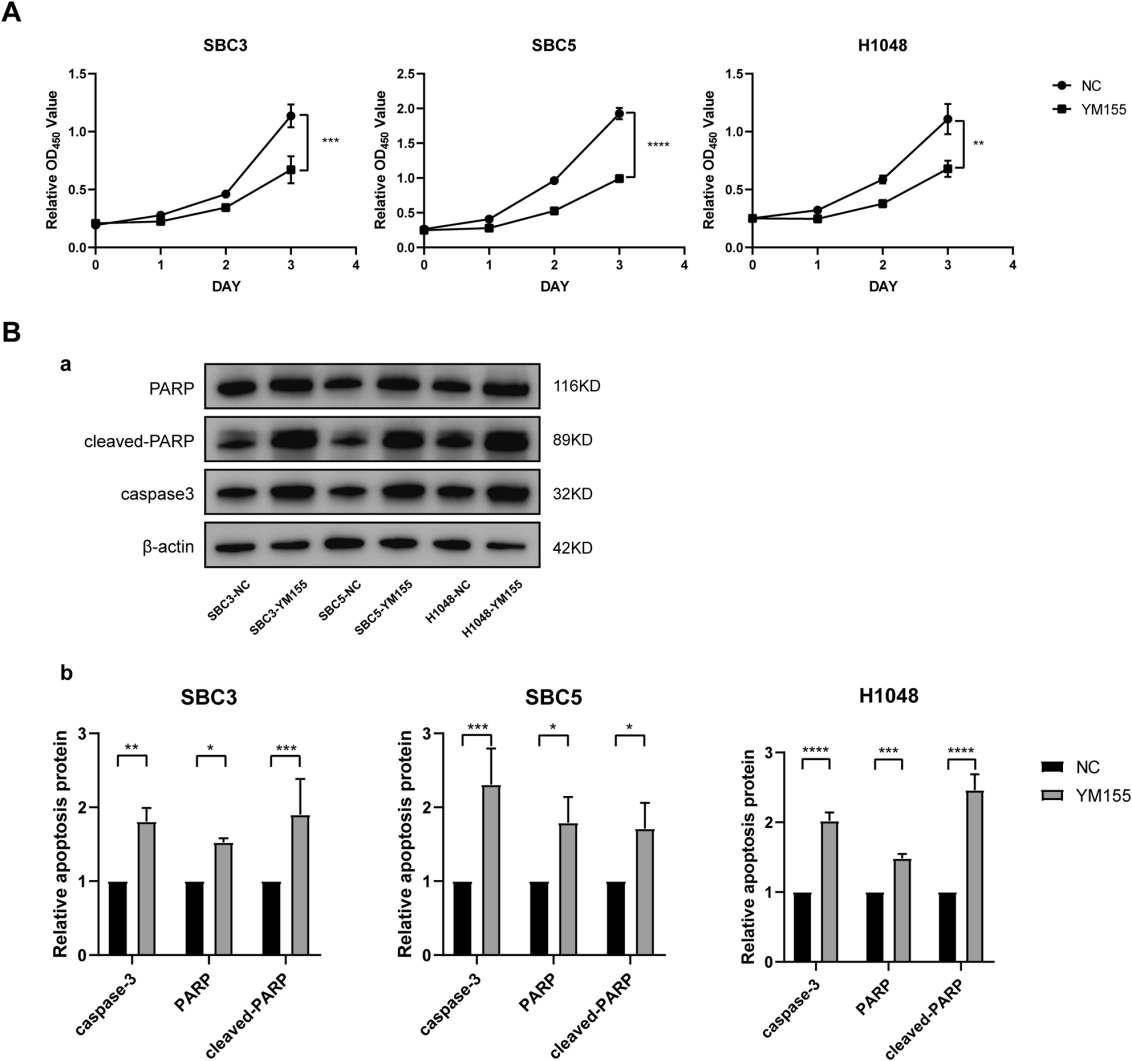
The application of BIRC5 inhibitor (YM155). (**A**) CCK8 cell proliferation assay results in SCLC cells after treatment with YM155. (**B**) The apoptosis detection after YM155 treatment was determined by western blotting (original blots are presented in Supplementary information 2).

Moreover, after treatment with BIRC5 inhibitor (YM155), the proliferation ability of SCLC cells decreased and apoptosis increased, which is consistent with the siRNA related experimental results (Fig. 7). These results indicate that BIRC5 indeed promotes the survival of SCLC cells.

## Discussion

In this study, we first analyzed potential biomarkers of SCLC using bioinformatics methods, and then evaluated their relationship with immune cell infiltration in SCLC. Next, we validated the results through some cell experiments.

In order to improve the accuracy of our findings, we screened DEGs using two ways. Then we identified seven genes (*AURKB, BIRC5, TOP2A, TYMS, PCNA, UBE2C, AURKA*) as our hub genes. These seven genes were expressed more strongly in SCLC samples than in healthy samples. Interestingly, all seven genes are believed to be involved in the occurrence or progression of lung cancer. Among them, DNA topoisomerase II alpha (TOP2A) is an important ribozyme in DNA metabolism, mainly involved in gene replication, cell cycle regulation, and other functions by reducing the DNA superhelix structure^15^. Studies have shown that TOP2A has a high level of expression in lung adenocarcinoma and is related to poor prognosis^16^. TOP2A had an inverse relationship with immune cells, including CD8 + T cells, eosinophils and natural killer (NK) cell^17^. In SCLC, cytotoxic drug etoposide can act on the TOP2A protein to prevent DNA replication and transcription^18^. However, a study of the relationship between SCLC and VP-19 resistance revealed that the decrease of expression of TOP2A may enhance drug resistance^19^. Aurora kinase A (AURKA) is a gene that codes for a kinase that regulates the cell cycle, and is believed to play a role in the development and maintenance of microtubules in the spindle pole during chromosomal separation^20^. AURKA is overexpressed in various cancers^21^. It paralyzes the G2/M checkpoint and spindle assembly checkpoint, leading to carcinogenic effects due to genomic instability^22,23^. Studies have shown that knocking down AURKA can cause mitotic arrest in the G2/M phase of SCLC cell lines H446 and H1688, and induce cell apoptosis^24^. A clinical study suggests that the AURKA inhibitor Alisertib could be also combined with paclitaxel as a second-line treatment for SCLC, and patients in the Alisertib/paclitaxel group have shown good efficacy^25^. AURKB (aurora kinase B) is a crucial serine/threonine kinase belonging to the protein kinase family. It is in charge of regulating cell mitosis and is important in the growth of tumors^26^. Research has shown that SCLC cell lines with RB1 deficiency (RB1 − / − SCLC cell line) were highly dependent on several proteins related to chromosome segregation, including Aurora B kinase. And RB1 − / − SCLC is sensitive more to Aurora B kinase inhibitors^27^. The thymidylate synthase (TYMS)is a key enzyme in the folate metabolism pathway and a target for many cytotoxic antifolate chemotherapy drugs, including 5-fluorouracil and capecitabine^28^. Research has found that TYMS appears to be associated with chemotherapy resistance in NSCLC, and its low expression may increase the sensitivity of NSCLC cells to pemetrexed^29^. Proliferating cell nuclear antigen (PCNA) is a protein present in all eukaryotic cells, closely related to DNA synthesis, and has a significant impact on initiating cell proliferation. It has been widely used as a biomarker for tumor progression in recent years^30^. Recently, researchers have developed a small molecule inhibitor of PCNA called AOH1996, which has the potential to be developed as a broad-spectrum anti-cancer drug. It can selectively inhibit tumor growth without causing significant side effects. This drug has currently entered the clinical trial stage^31^. Ubiquitin conjugating enzyme E2C (UBE2C) has been proven to be a pro-cancer factor in multiple studies^32–34^. In NSCLC, its inhibitory effect on autophagy of cancer cells is closely related to tumor cell growth and malignant phenotype. Research has indicated that UBE2C may facilitate the occurrence and progression of Kras^G12D^ lung cancer, and may become a new target for Kras^G12D^ lung cancer^35^.

As the GO analysis results for DEGs showed that they were related to immune functions, such as “cell chemotaxis”, “leukocyte chemotaxis”, and “leukocyte migration”, we decided to perform an immune infiltration analysis on all samples. We found that seven types of immune cells may be related to immune infiltration in SCLC, so we next studied the connection between these seven immune cells and hub genes, to identify genes that might have an impact on the immune microenvironment of SCLC. Finally, we found that the content of monocytes and BIRC5 expression were negatively correlated. BIRC5 (Survivin) is a member of a family of proteins that are inhibitors of apoptosis with tumor specificity, generally expressed in tumor and embryonic tissues. Studies have shown that BIRC5 is over expressed in over 60 types of tumors and is strongly linked to the differentiation, proliferation, infiltration, and metastasis of tumor cells^36,37^. In studies on the relationship between BIRC5 and colorectal tumors, the BIRC5 expression sequentially increased in low-grade dysplastic adenomas, highly dysplastic adenomas, and adenocarcinoma tissues, which may be related to the malignant transformation of colorectal tumors^38^. Furthermore, in researches of patients with breast cancer, lymphoid leukemia and melanoma, BIRC5 can be recognized by cytotoxic T lymphocytes and generate immune response^39^. BIRC5 can also promote tumor resistance to broad-spectrum chemotherapy drugs, radiation insensitivity, and lead to poor prognosis^40,41^. Given the importance of BIRC5 in tumors, many clinical studies on small molecule inhibitors targeting it are ongoing. Although some of these inhibitors have shown certain therapeutic effects, off target effects often occur^40^. It is gratifying that immunotherapy based on BIRC5 is gradually receiving attention, and some survivin vaccines have entered clinical research, which may provide new options for cancer treatment in the future^42^. In lung cancer, research into BIRC5 has mainly concentrated on NSCLC. Compared with normal tissue, NSCLC tissue had a considerably higher level of BIRC5 expression, which was linked to infiltration, metastasis, and prognosis^43^. Another study showed that the BIRC5 protein was strongly expressed in NSCLC afatinib resistant cell lines, and the use of BRCA5 inhibitor YM155 can lead to increased NSCLC sensitivity to afatinib^44^. However, clinical trials of combining YM155 with chemotherapy in NSCLC have not yet yielded positive results^45,46^. Studies of the role of BIRC5 in SCLC are few and controversial. Studies have reported that although over expression of BIRC5 has an impact on the prognosis of NSCLC, it is not directly related to the prognosis of SCLC^47^. But another study concluded that high expression of BIRC5 in SCLC may indicate poor prognosis^48^.

According to our bioinformatics analysis results, among these seven hub genes, only BIRC5 is significantly correlated with SCLC related immune cells. We decided to conduct cell experiments to verify the relationship between BIRC5 and SCLC. Consistent with the bioinformatics analysis results, cell experiments showed higher expression of BIRC5 in all three SCLC cell lines than in HLF. It is interesting to note that BIRC5 expression in SCLC cells was also higher than in NSCLC cells. Subsequent in vitro experimental results showed that BIRC5 can be vital in SCLC through regulating cell viability, inhibiting cell apoptosis, and promoting cell invasion and migration. The experimental results using BIRC5 inhibitor YM155 also support this conclusion.

Our research has limitations. Firstly, we conducted in vitro experiments only. In the future, we should conduct in vivo experiments to further validate the analysis results. Furthermore, we note that our previous analysis of the SCLC immune microenvironment showed a negative correlation between BIRC5 and monocyte content. However, researches have revealed that SCLC patients treated with ICIs usually have better prognoses when their lymphocyte to monocyte ratio (LMR) is higher^49^. This indicates that the role of BIRC5 in the immune microenvironment of SCLC may be more complex than the inhibitory effect of BIRC5 on cell apoptosis and the promotion of invasion in SCLC. Meanwhile, depending on the stage of tumor development, different monocyte subsets exhibit different or even opposite effects. On the one hand, monocytes can inhibit tumor growth by phagocytosing and killing tumor cells. On the other hand, they can also promote tumor growth and lead to metastasis by secreting various cytokines^50^. The role of monocytes in tumor immunity requires further exploration. In summary, the relationship between BIRC5 and monocytes should be further validated and studied through experiments.

## Conclusion

In summary, we identified AURKB, BIRC5, TOP2A, TYMS, PCNA, UBE2C, and AURKA genes as potential biomarkers for SCLC. The high expression of BIRC5 promotes the development of SCLC and may contribute to changes in the immune microenvironment of SCLC by affecting monocytes.

### Supplementary Information

Supplementary Information 1.

Supplementary Information 2.

Supplementary Information 3.

Supplementary Information 4.

Supplementary Information 5.

Supplementary Information 6.

## Data Availability

The materials described in the manuscript, including all relevant raw data, will be provided free of charge by the corresponding author to any researcher who wishes to use them for non-commercial purposes, without violating the confidentiality of the participants.
