# Supplementary material for: Exploring effective biomarkers and potential immune related gene in small cell lung cancer
Source: Sci Rep. 2024 Mar 31;14:7604. doi: 10.1038/s41598-024-58454-4 (PMC10982305; doi:10.1038/s41598-024-58454-4)
Supplement: Supplementary file 5 — Supplementary Information 5. [file 41598_2024_58454_MOESM5_ESM.pdf]

Supplementary Legends and Figures

Figure S1. Analysis of the pathways and functionality of DEGs. (A, B) Barplot and cnetplot of KEGG pathway enrichment analysis results. (C, D) Barplot and cnetplot of GO functional enrichment analysis results.

Figure S2. PPI network. Red represents upregulated genes, blue represents downregulated genes.

Figure S3. The violin plots showed the differential expression of hub genes in GSE11969. The comparison between the two sets of data was conducted using the mean t-test. P-value <0.05 is considered statistically significant.

Figure S4. Identification of significantly different infiltrating immune cells in SCLC samples. (A) Wilcoxon test results showed 13 types of immune cells. (B, C) Lasso regression results showed 7 types of immune cells. (D) Two different methods obtained 7 Intersecting immune cells.

Figure S5. BIRC5 inhibited the apoptosis of SCLC cells and promoted their proliferation, migration and invasion. (A) Cell apoptosis detected by Flow cytometry (a) and western blot (b). (B) Scratch wound healing assay results. (C) Transwell cell invasion assay results.

Figure S1.

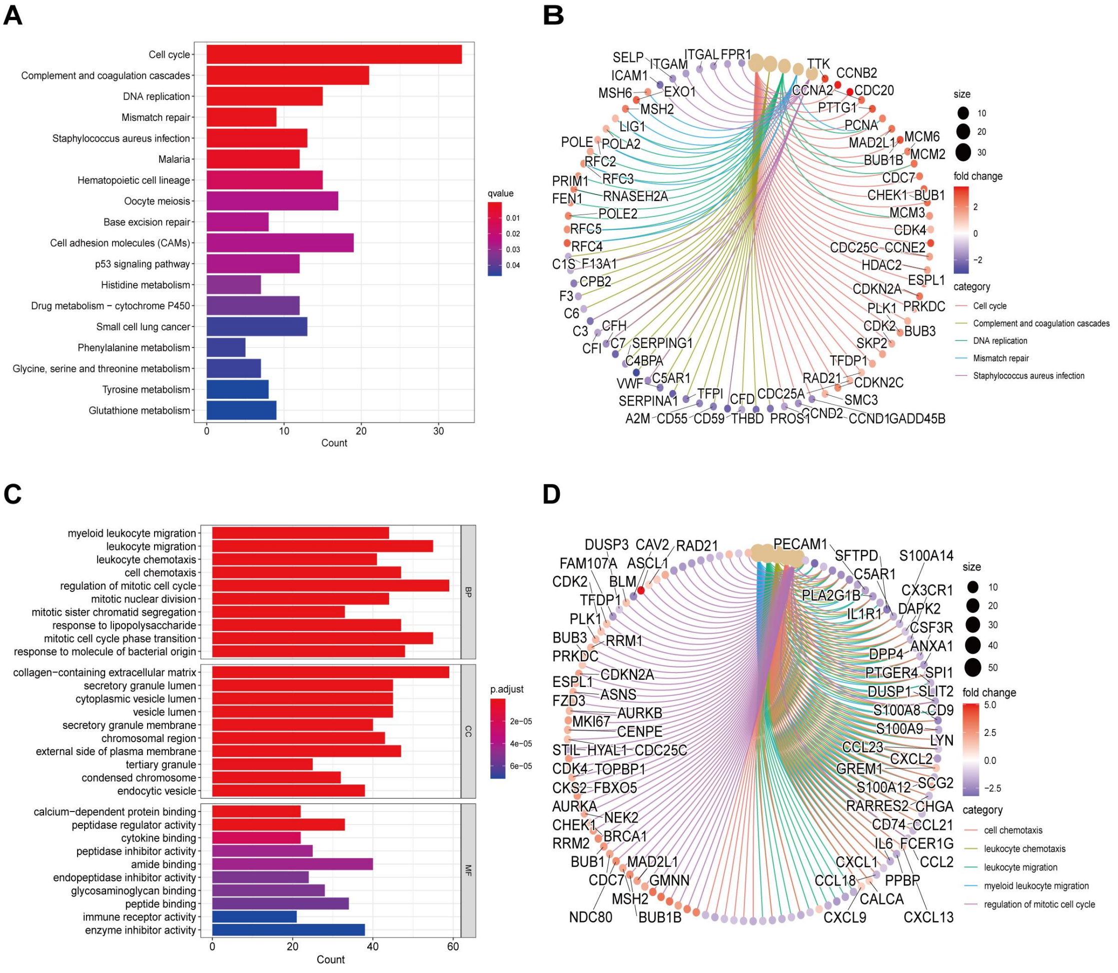
A
Cell cycle
Complement and coagulation cascades
DNA replication
Mismatch repair
Staphylococcus aureus infection
Malaria
Hematopoietic cell lineage
Oocyte meiosis
Base excision repair
Cell adhesion molecules (CAMs)
p53 signaling pathway
Histidine metabolism
Drug metabolism − cytochrome P450
Small cell lung cancer
Phenylalanine metabolism
Glycine, serine and threonine metabolism
Tyrosine metabolism
Glutathione metabolism
Count
qvalue
B
size
fold change
category
Cell cycle
Complement and coagulation cascades
DNA replication
Mismatch repair
Staphylococcus aureus infection
C
myeloid leukocyte migration
leukocyte migration
leukocyte chemotaxis
cell chemotaxis
regulation of mitotic cell cycle
mitotic nuclear division
mitotic sister chromatid segregation
response to lipopolysaccharide
mitotic cell cycle phase transition
response to molecule of bacterial origin
collagen−containing extracellular matrix
secretory granule lumen
cytoplasmic vesicle lumen
vesicle lumen
secretory granule membrane
chromosomal region
external side of plasma membrane
tertiary granule
condensed chromosome
endocytic vesicle
calcium−dependent protein binding
peptidase regulator activity
cytokine binding
peptidase inhibitor activity
amide binding
endopeptidase inhibitor activity
glycosaminoglycan binding
peptide binding
immune receptor activity
enzyme inhibitor activity
BP
CC
MF
Count
p.adjust
D
size
fold change
category
cell chemotaxis
leukocyte chemotaxis
leukocyte migration
myeloid leukocyte migration
regulation of mitotic cell cycle

Figure S2.

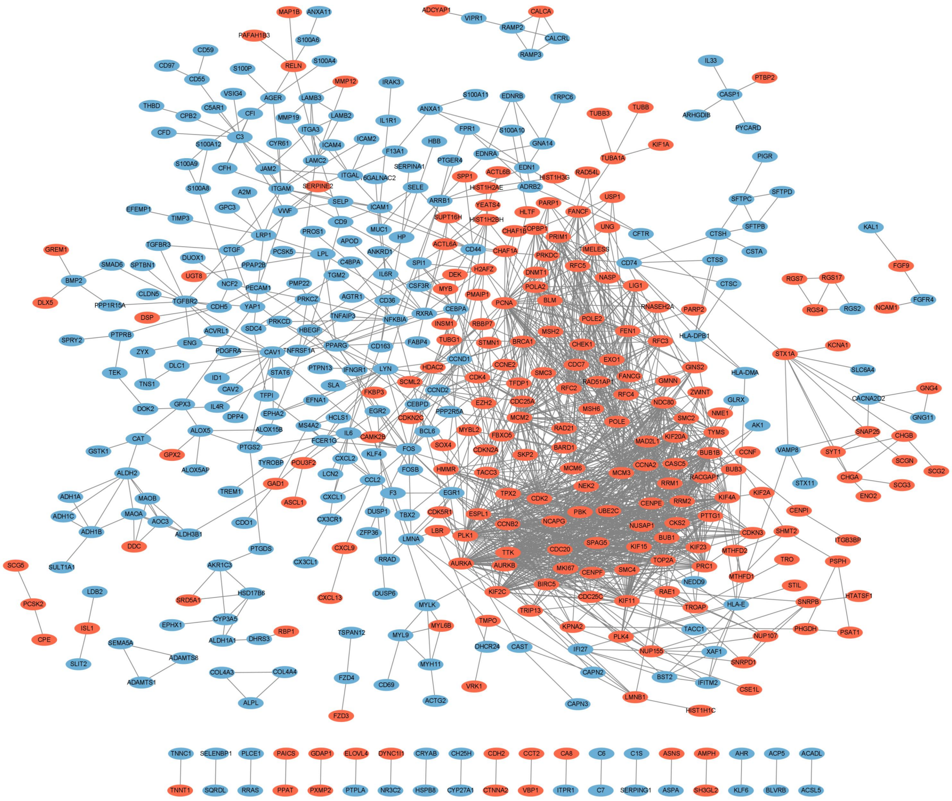

Figure S3.

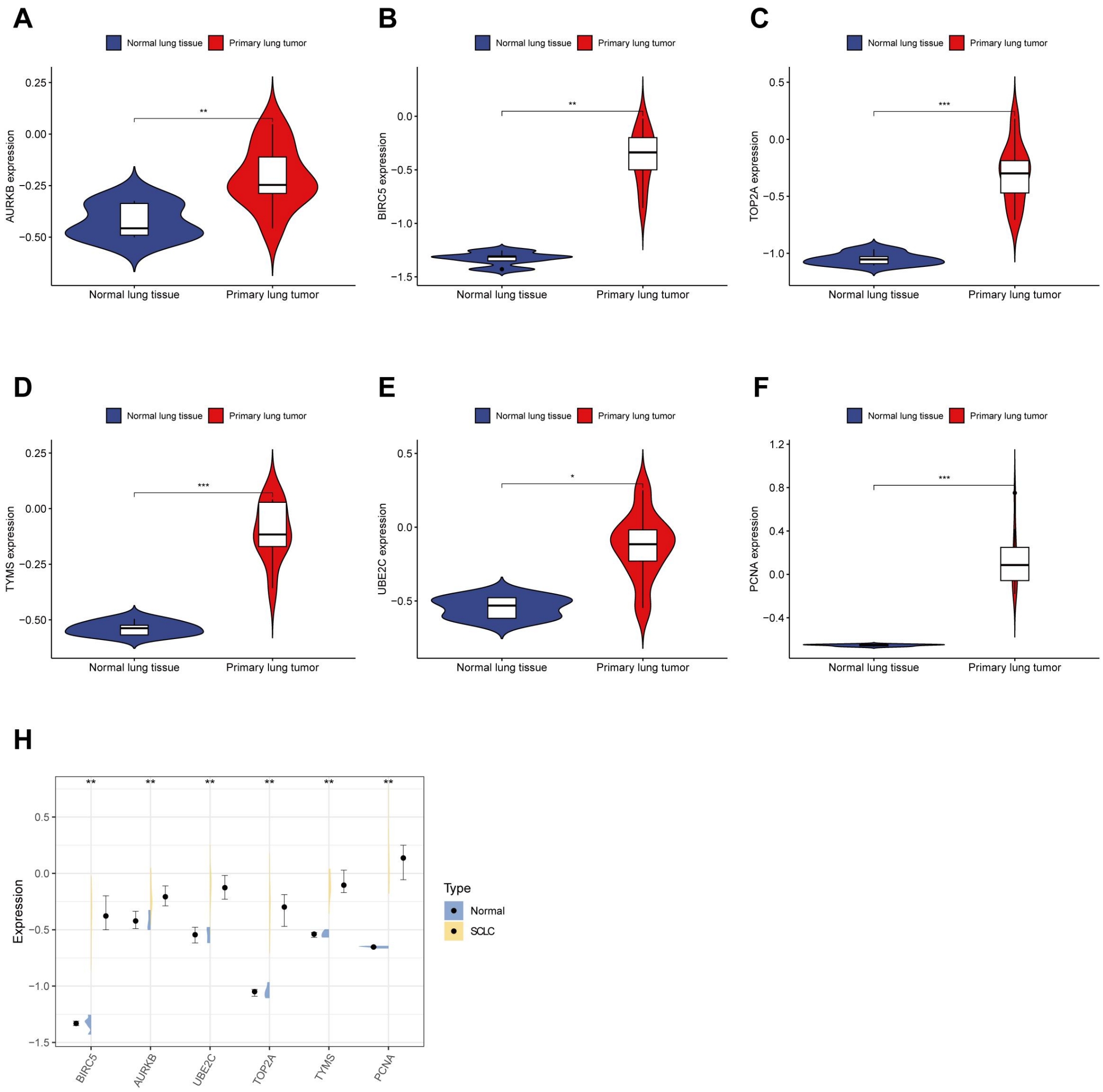
A
B
C
D
E
F
H
Normal lung tissue
Primary lung tumor
AURKB expression
BIRC5 expression
TOP2A expression
TYMS expression
UBE2C expression
PCNA expression
Expression
Type
Normal
SCLC
BIRC5
AURKB
UBE2C
TOP2A
TYMS
PCNA

Figure S4

A

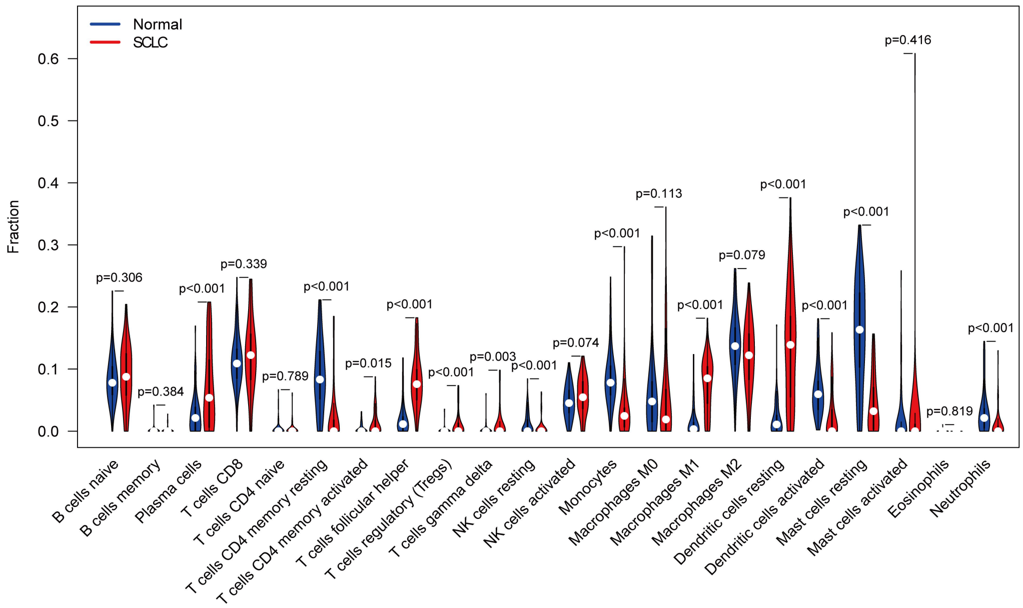

B

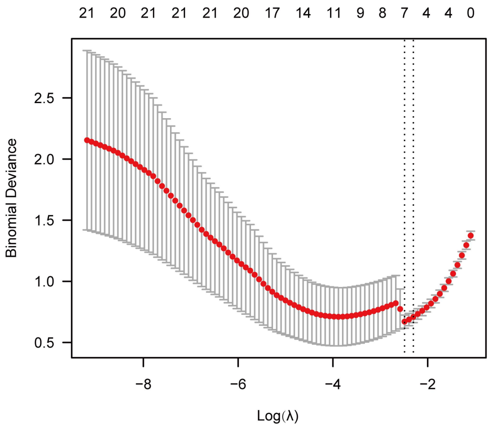

C

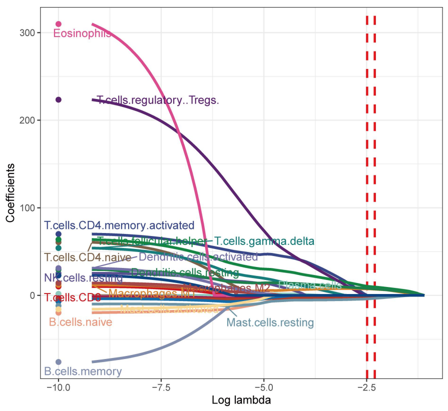

D

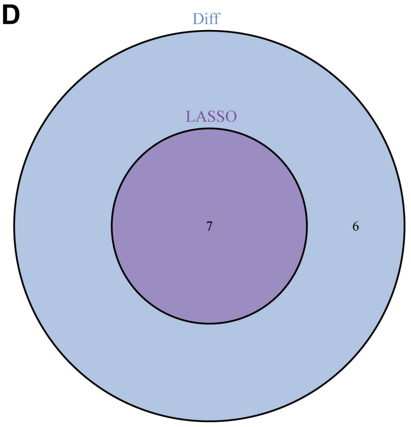

Figure S5.

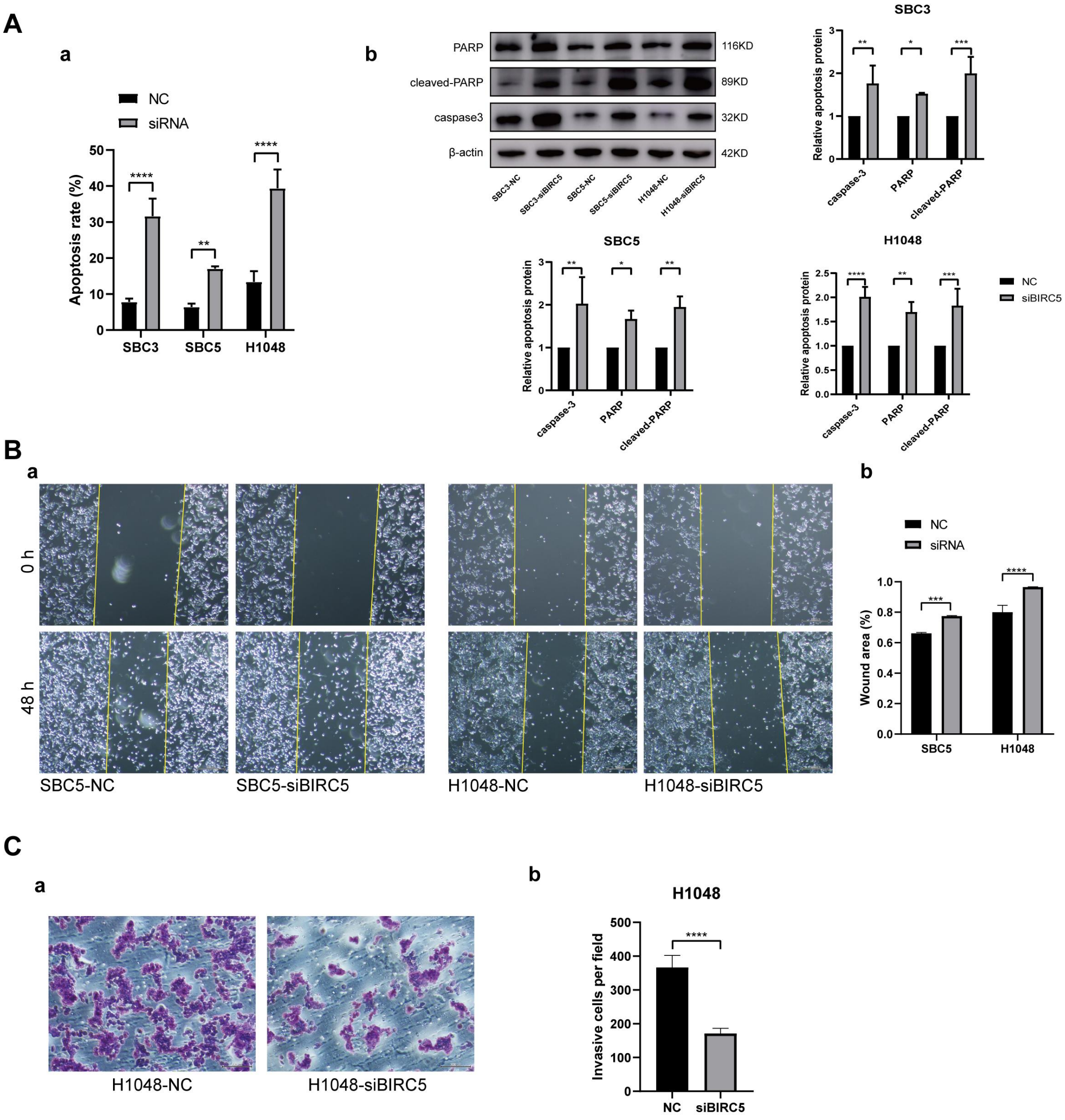
A
a
NC
siRNA
Apoptosis rate (%)
SBC3
SBC5
H1048
b
PARP
cleaved-PARP
caspase3
β-actin
116KD
89KD
32KD
42KD
SBC3-NC
SBC3-siBIRC5
SBC5-NC
SBC5-siBIRC5
H1048-NC
H1048-siBIRC5
SBC3
SBC5
H1048
Relative apoptosis protein
caspase-3
PARP
cleaved-PARP
NC
siBIRC5
B
a
0 h
48 h
SBC5-NC
SBC5-siBIRC5
H1048-NC
H1048-siBIRC5
b
NC
siRNA
Wound area (%)
SBC5
H1048
C
a
H1048-NC
H1048-siBIRC5
b
H1048
Invasive cells per field
NC
siBIRC5

Table S1. 7 intersecting immune cells obtained by Wilcoxon test and LASSO regression

T cells CD4 memory resting
T cells CD4 memory activated
T cells follicular helper
Monocytes
Macrophages M1
Dendritic cells resting
Mast cells resting
