# Supplementary material for: Exploring effective biomarkers and potential immune related gene in small cell lung cancer
Source: Sci Rep. 2024 Mar 31;14:7604. doi: 10.1038/s41598-024-58454-4 (PMC10982305; doi:10.1038/s41598-024-58454-4)

Original images of western blots:
Statement: All the blots were cut prior to incubation with antibodies.

Figure 6B: Verification of BIRC5 RNA knockout effect in SCLC cells by western blotting

β-actin

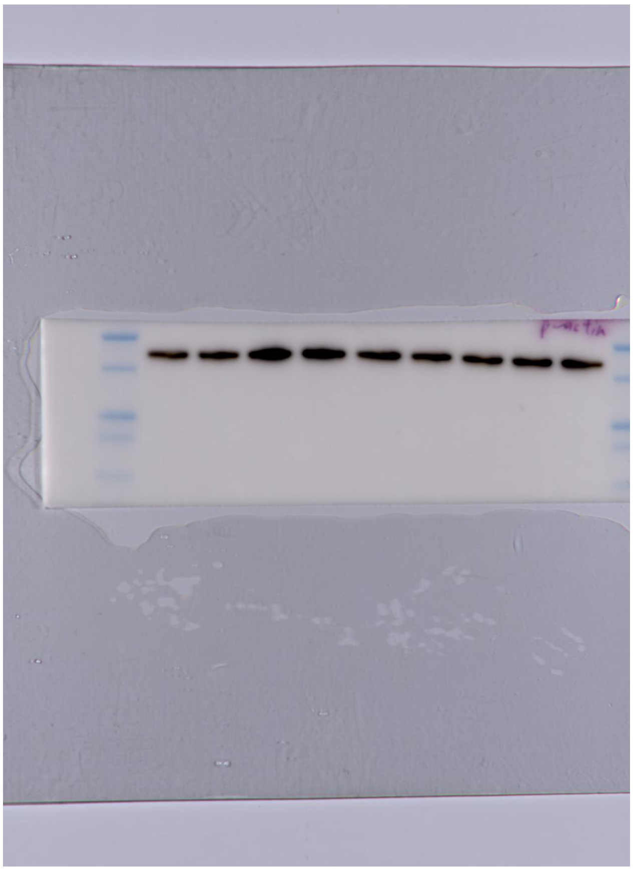

loading order (From Left To Right): SBC3-NC、SBC3-BIRC5 siR 1#、SBC3-BIRC5 siR 2#、SBC5-BIRC5 NC、SBC5-BIRC5 siR 1#、SBC5-BIRC5 siR 2#、H1048-NC、H1048-BIRC5 siR 1#、H1048-BIRC5 siR 2#.

BIRC5

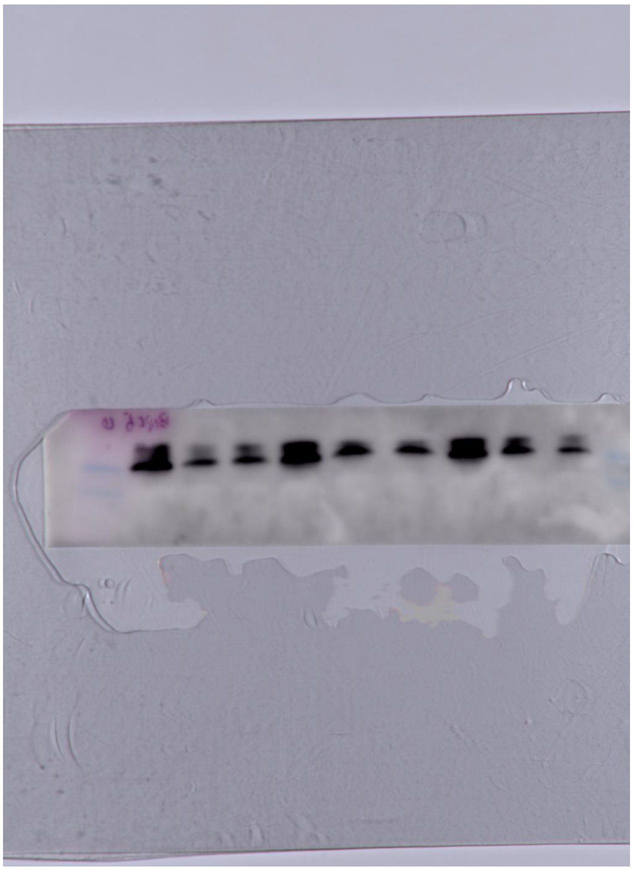

loading order (From Left To Right): SBC3-NC、SBC3-BIRC5 siR 1#、SBC3-BIRC5 siR 2#、SBC5-BIRC5 NC、SBC5-BIRC5 siR 1#、SBC5-BIRC5 siR 2#、H1048-NC、H1048-BIRC5 siR 1#、H1048-BIRC5 siR 2#.

Figure 7B The apoptosis protein detection of SCLC cells after YM155 treatment was determined by western blotting

β-actin

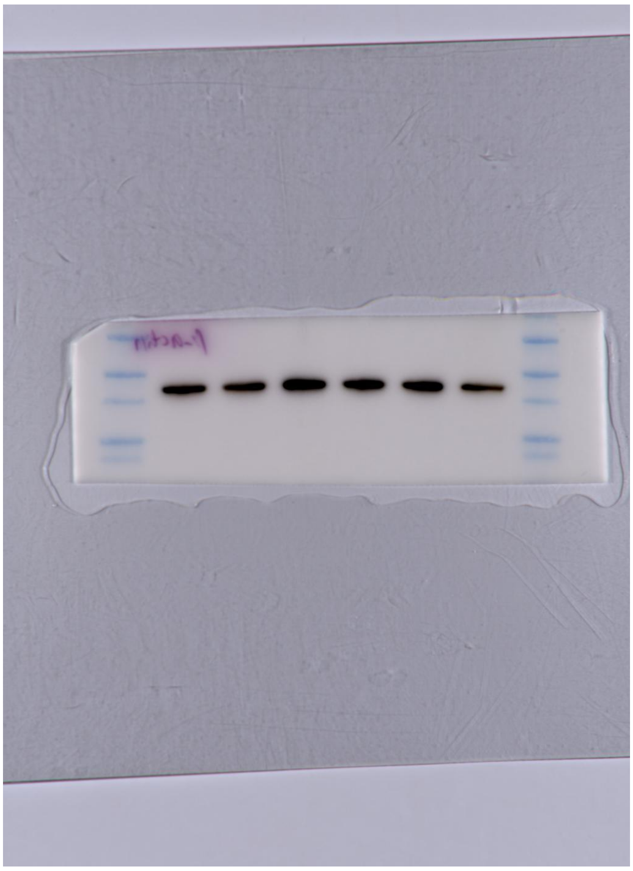

loading order (From Left To Right): SBC3-NC、SBC3-YM155、SBC5-NC、SBC5-YM155、H1048-NC、H1048-YM155.

Caspase-3

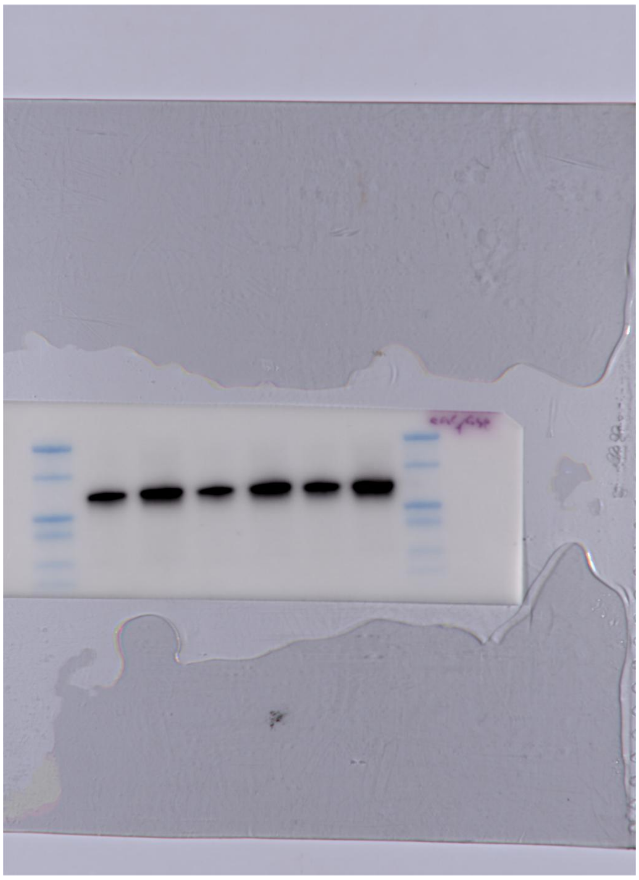

loading order (From Left To Right): SBC3-NC、SBC3-YM155、SBC5-NC、SBC5-YM155、H1048-NC、H1048-YM155.

Cleaved-PARP

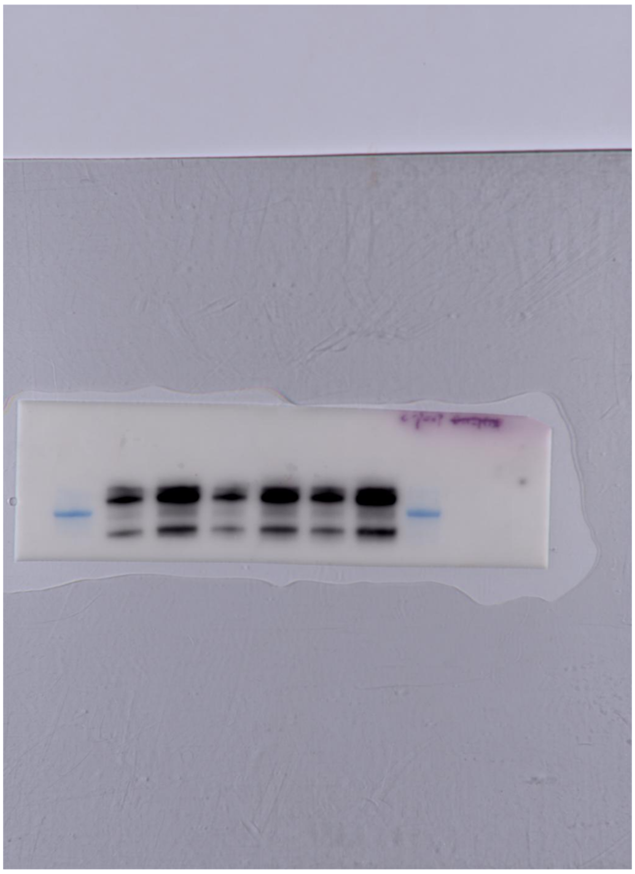

loading order (From Left To Right): SBC3-NC、SBC3-YM155、SBC5-NC、SBC5-YM155、H1048-NC、H1048-YM155.

PARP

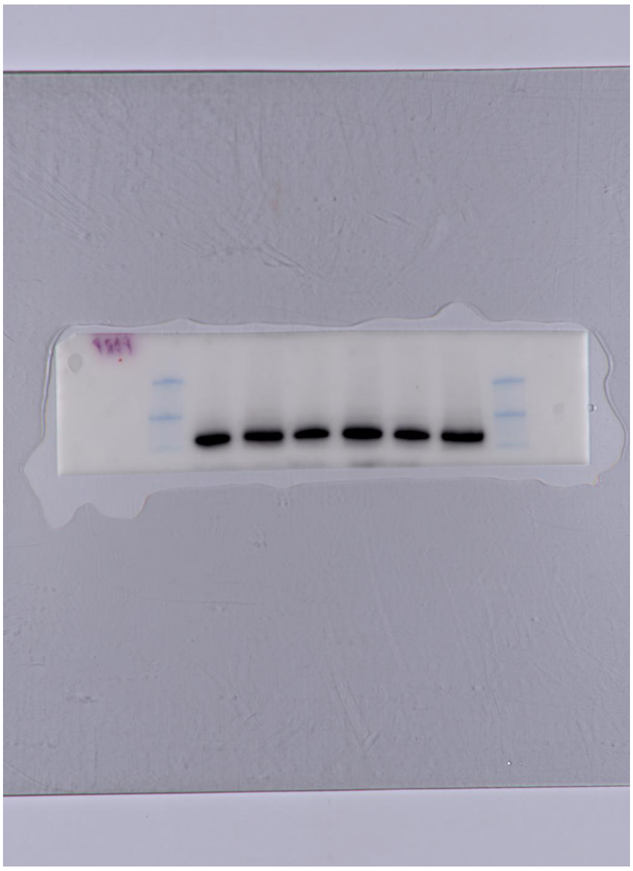

loading order (From Left To Right): SBC3-NC 、 SBC3-YM155 、 SBC5-NC 、 SBC5-YM155、H1048-NC、H1048-YM155.

Figure S5A. Cell apoptosis protein detected by western blot after BIRC5 RNA downregulation in SCLC cells.

β-actin

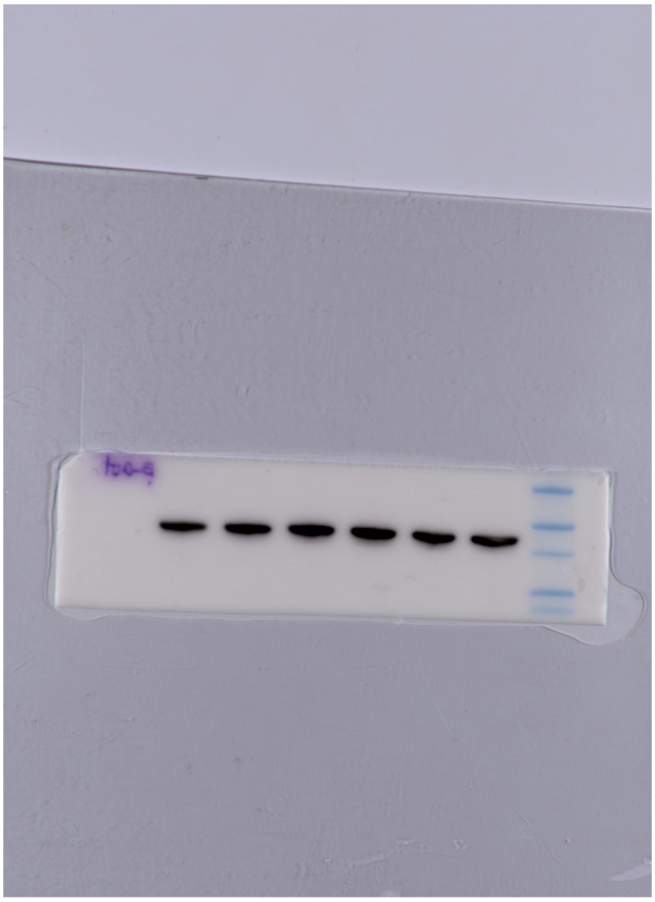

loading order (From Left To Right): SBC3-NC、SBC3-siBIRC5、SBC5-NC、SBC5-siBIRC5、H1048-NC、H1048-siBIRC5.

Caspase-3

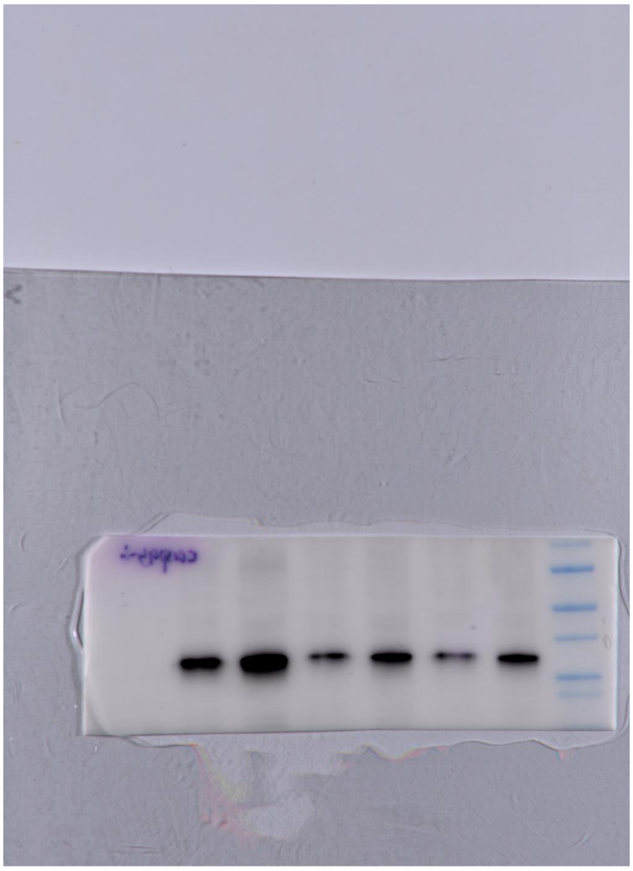

loading order (From Left To Right): SBC3-NC、SBC3-siBIRC5、SBC5-NC、SBC5-siBIRC5、H1048-NC、H1048-siBIRC5.

Cleaved-PARP

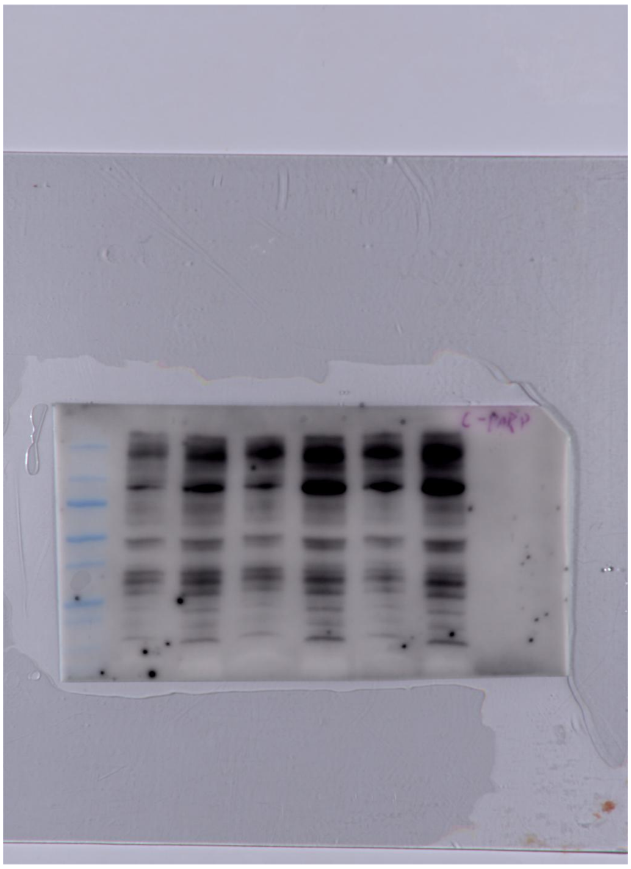

loading order (From Left To Right): SBC3-NC、SBC3-siBIRC5、SBC5-NC、SBC5-siBIRC5、H1048-NC、H1048-siBIRC5.

PARP

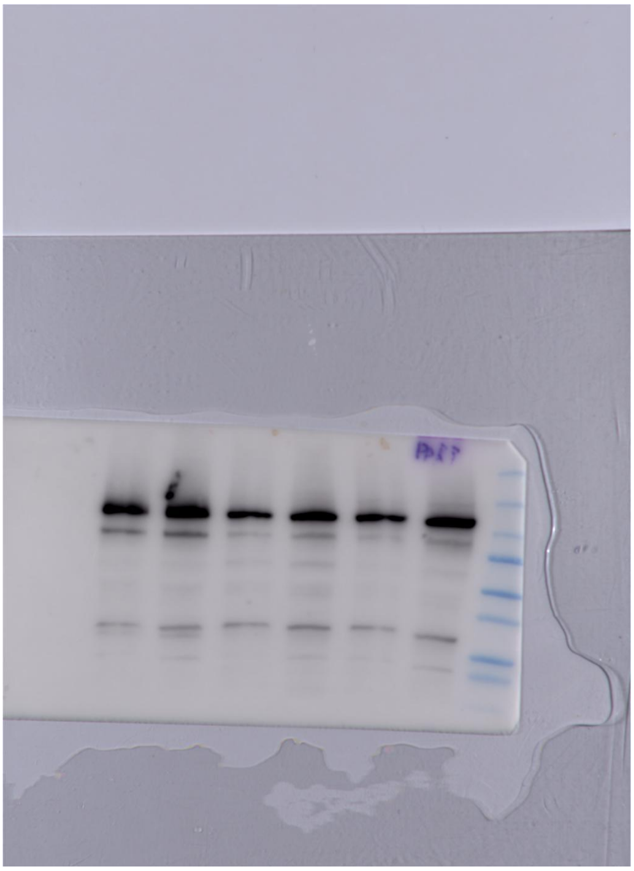

loading order (From Left To Right): SBC3-NC、SBC3-siBIRC5、SBC5-NC、SBC5-siBIRC5、H1048-NC、H1048-siBIRC5.

Original images of Scratch wound healing assay results:

Figure 6B: Scratch wound healing assay results

SBC3-NC
0h

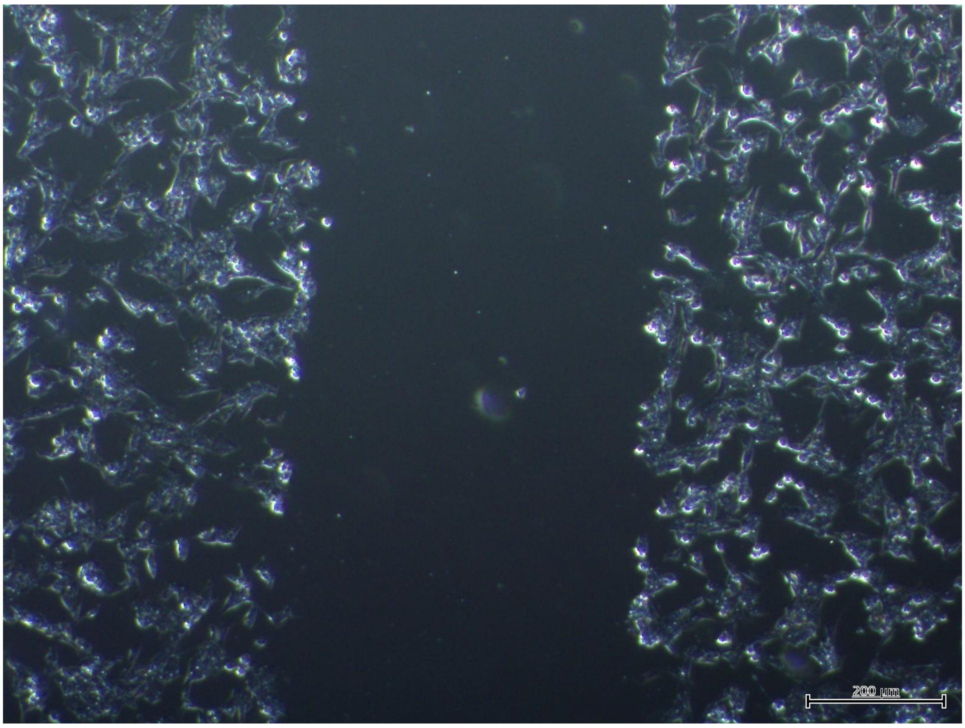

48h

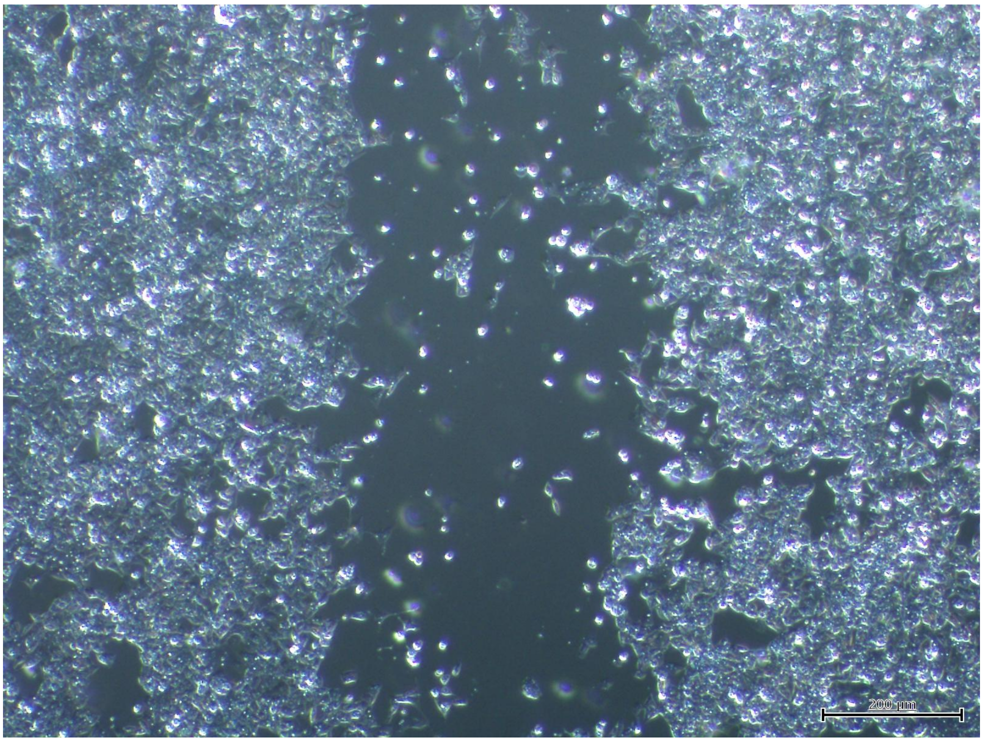

SBC3-siBIRC5

0h

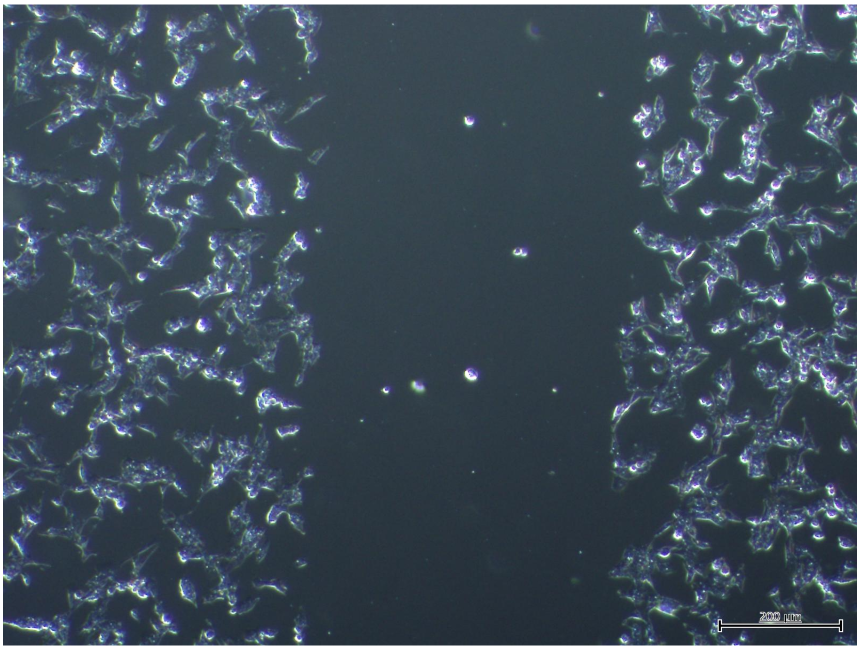

48h

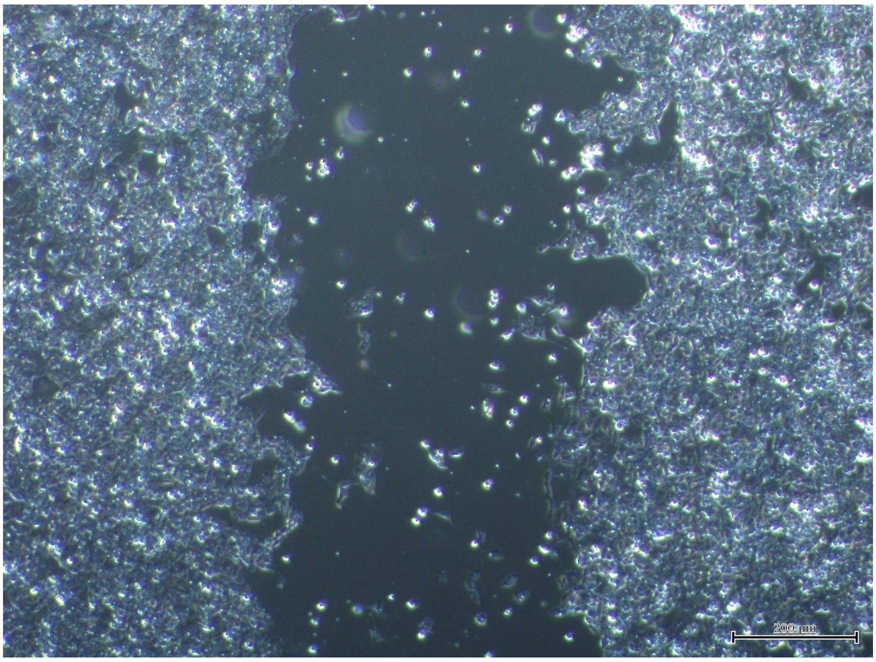

Figure S5B: **Scratch wound healing assay results**

SBC5-NC

0h

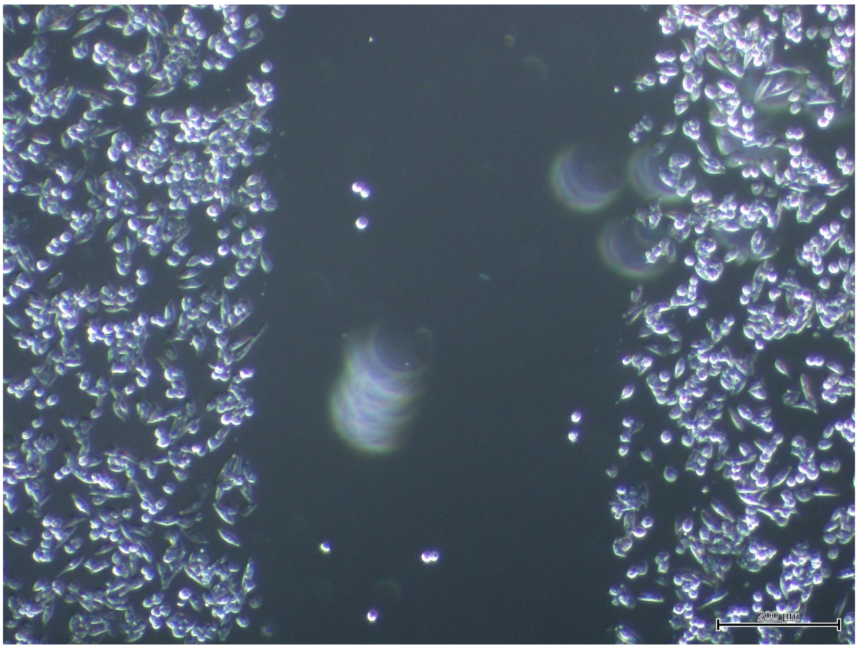

48h

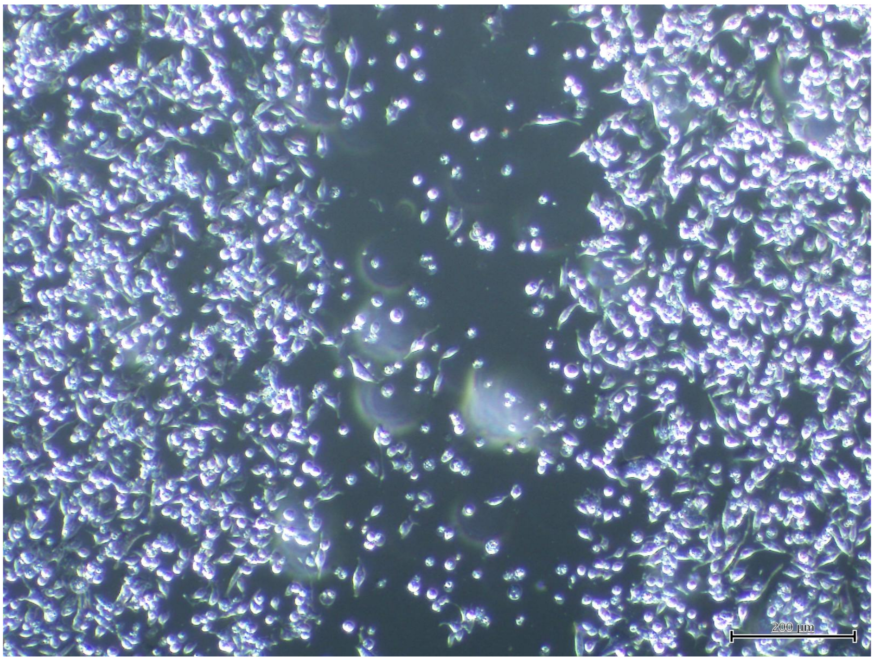

SBC5-siBIRC5

0h

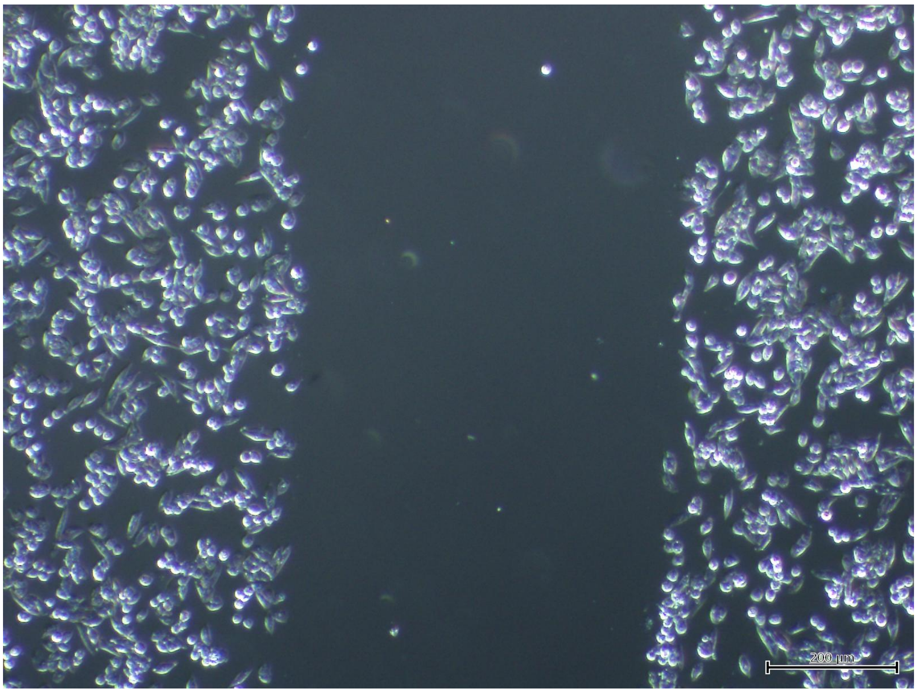

48h

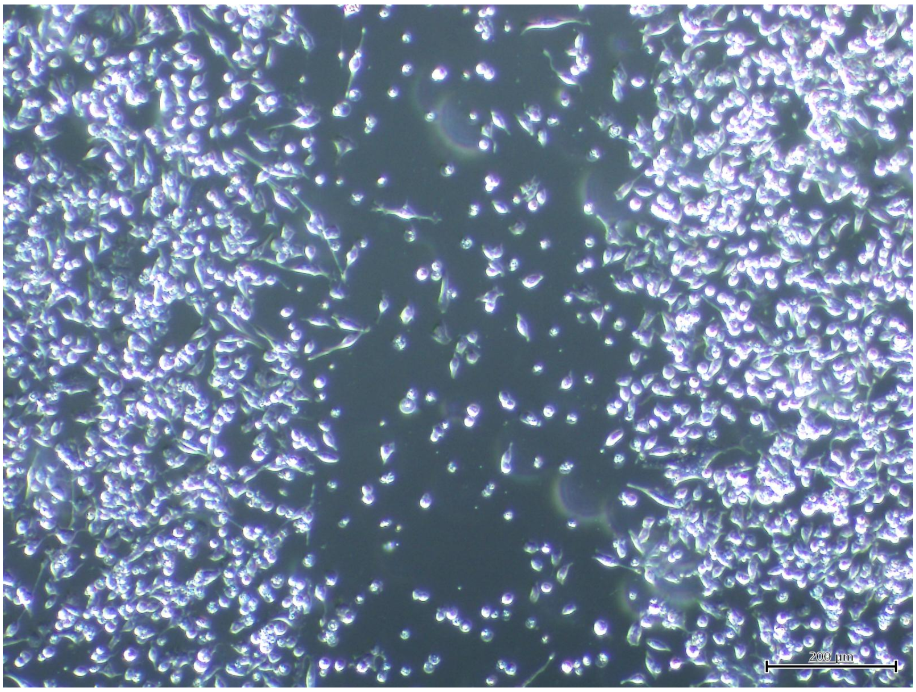

H1048-NC

0h

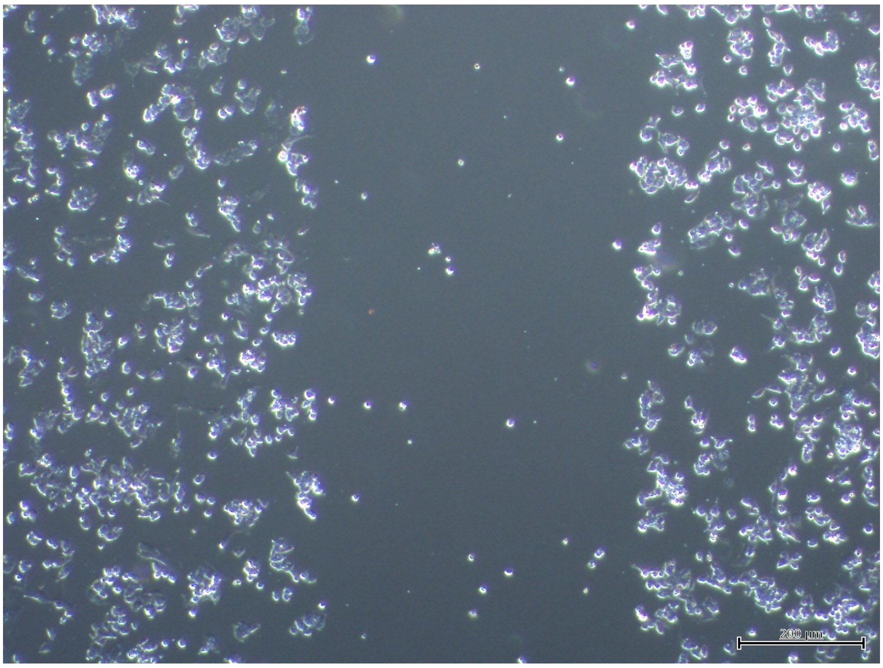

48h

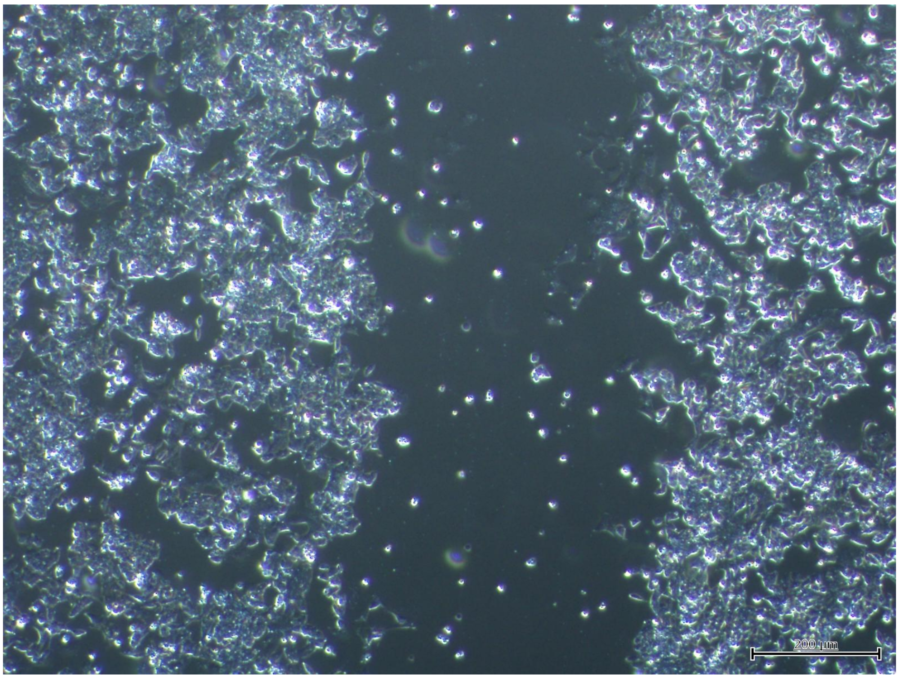

H1048-siBIRC5

0h

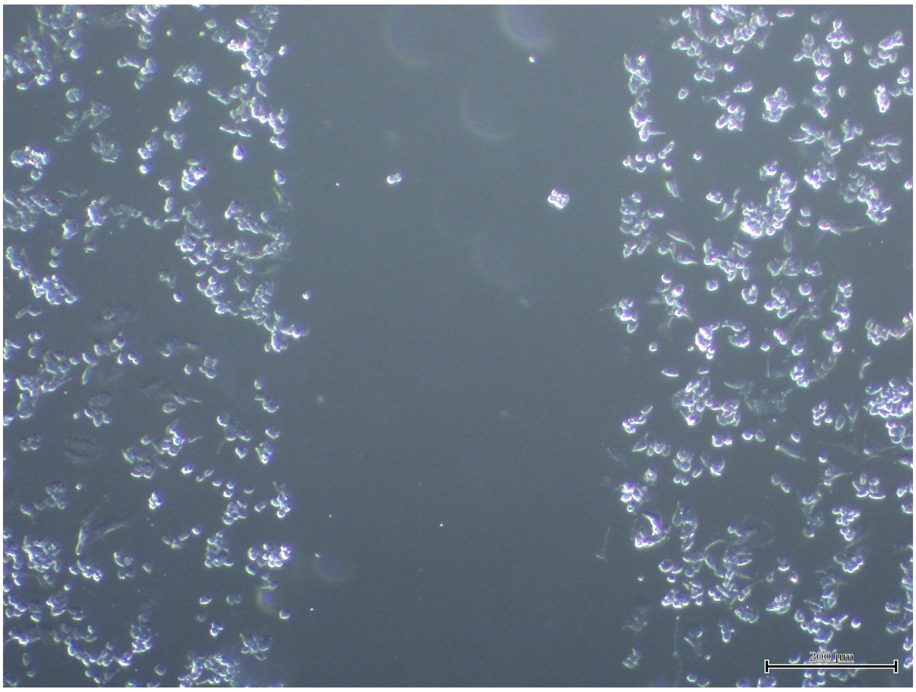

48h

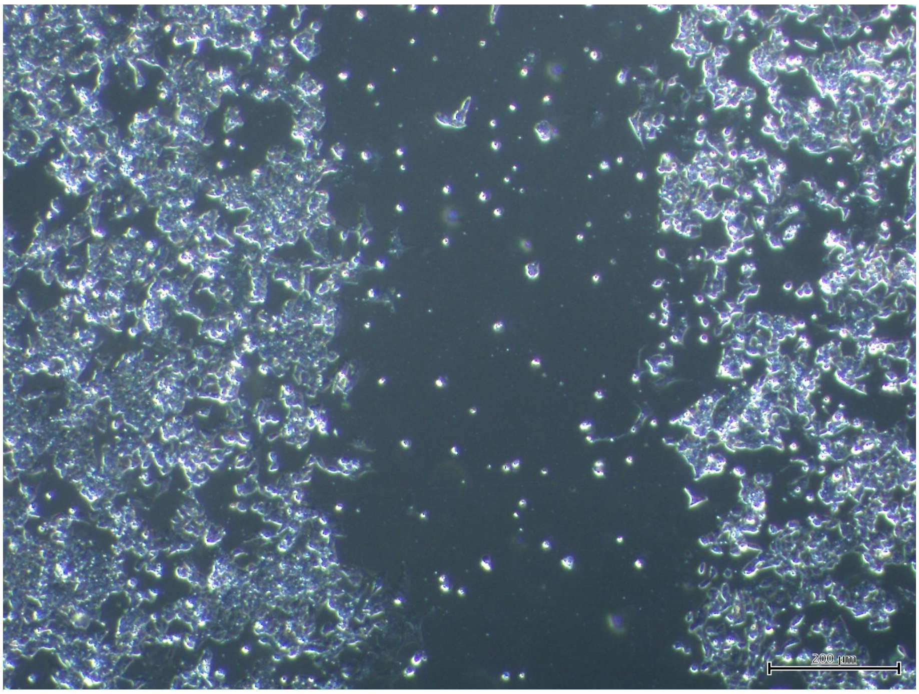

Original images of Transwell cell invasion assay results:

Figure 6F: Transwell cell invasion assay results

SBC3-NC

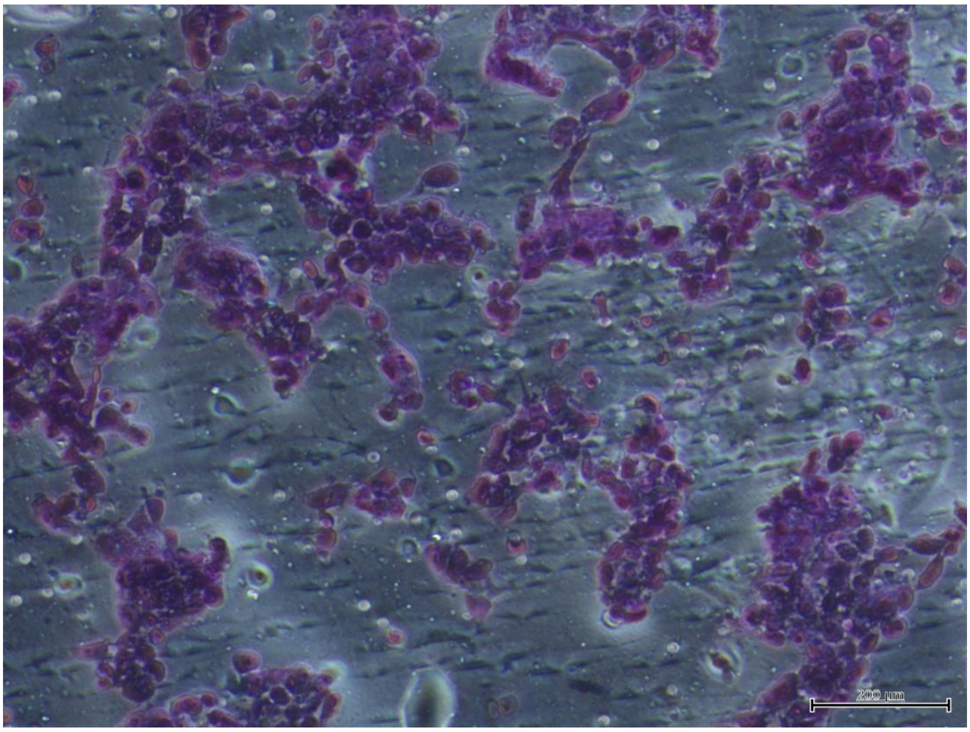

SBC3-BIRC5

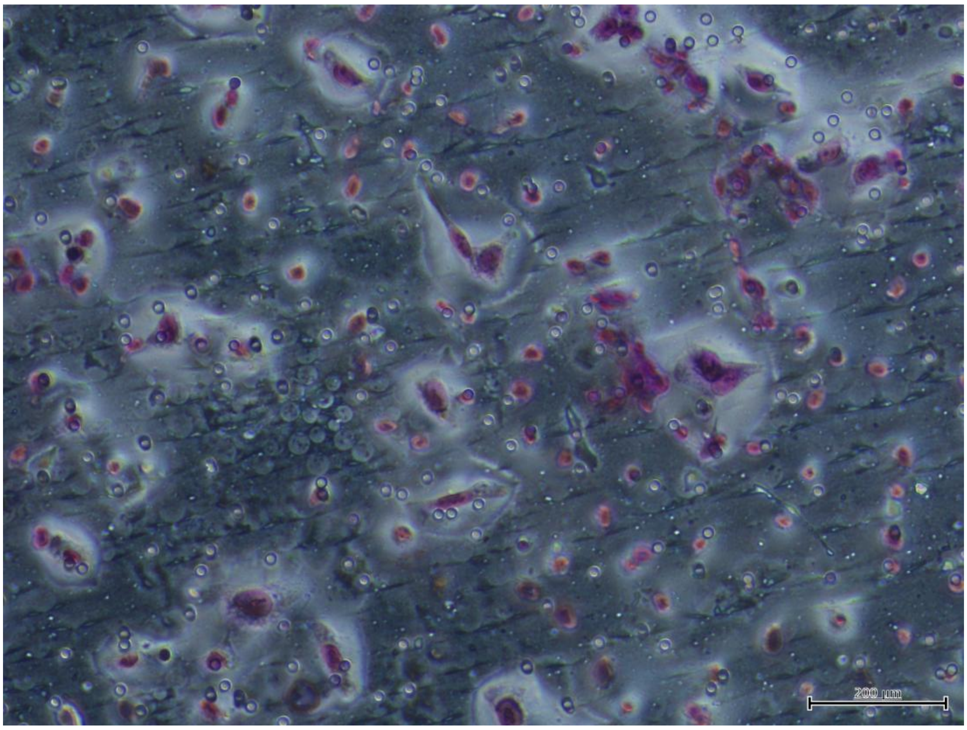

SBC5-NC

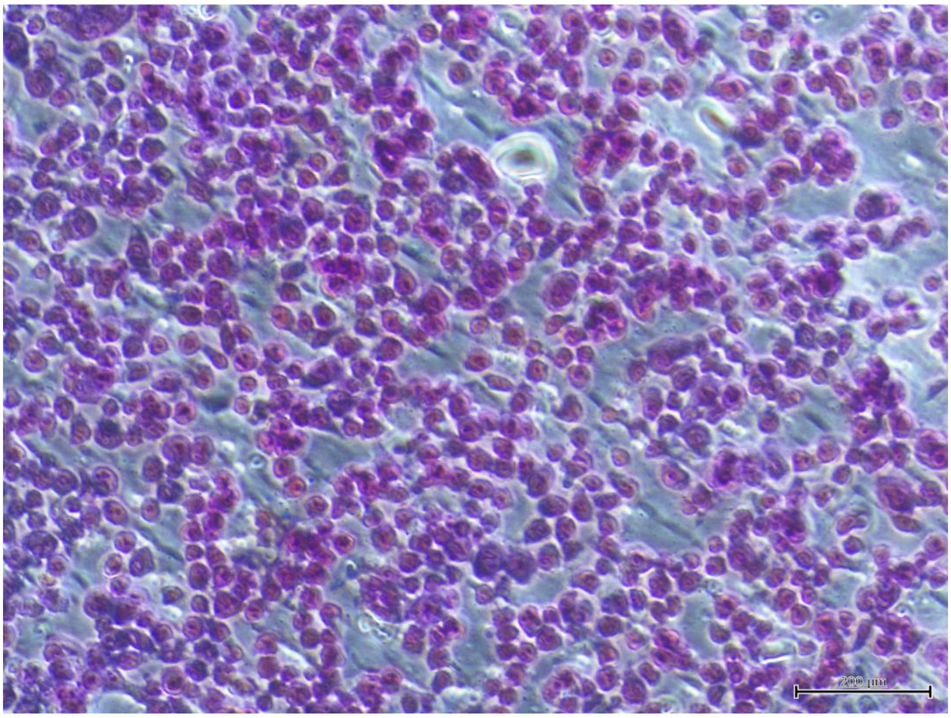

SBC5-BIRC5

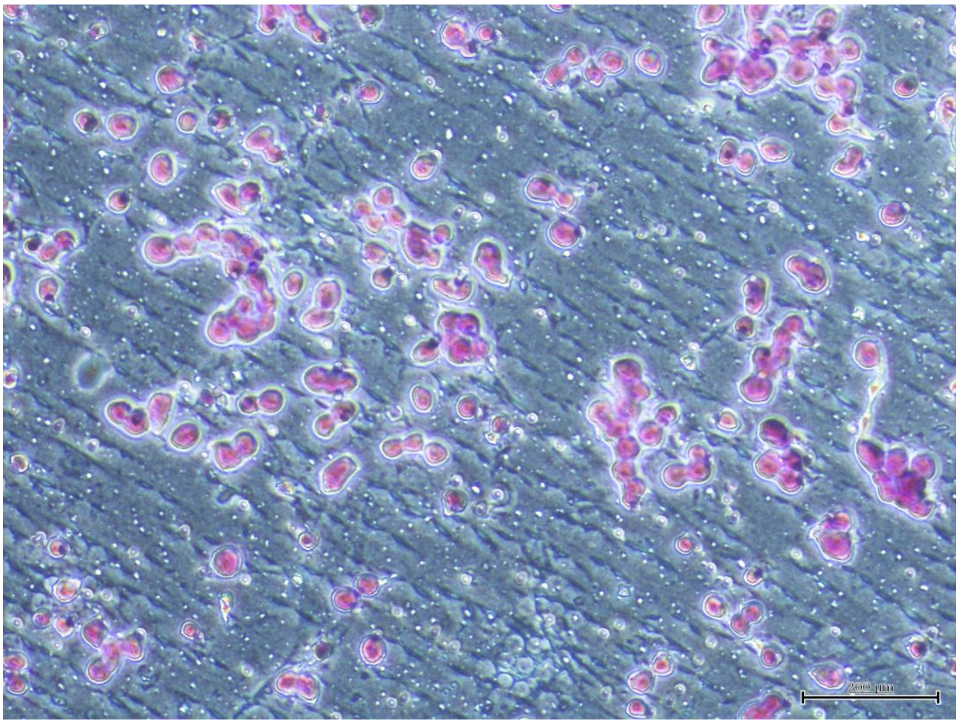

Figure S5C: **Transwell cell invasion assay results**

H1048-NC

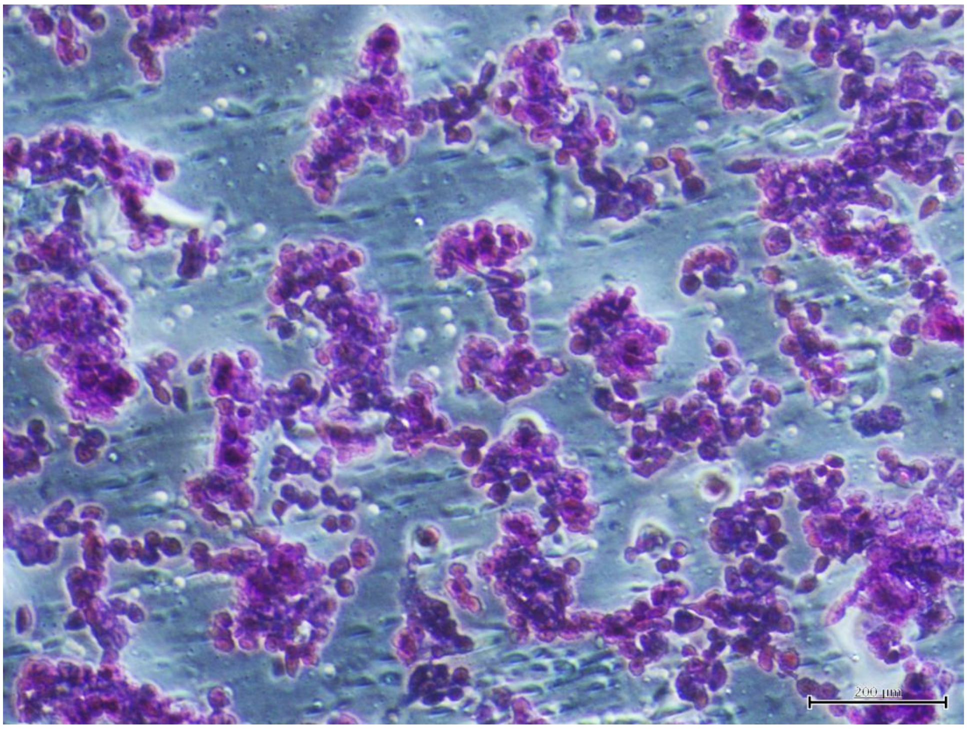

H1048-BIRC5

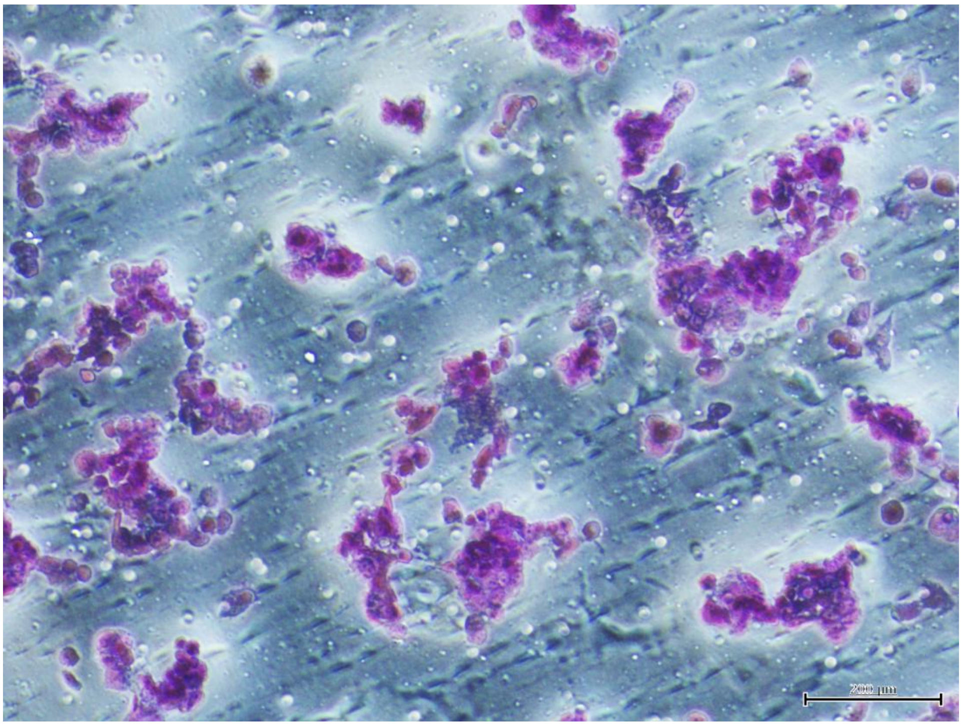

Supplement: Supplementary file 6 — Supplementary Information 6. [file 41598_2024_58454_MOESM6_ESM.pdf]
